# Synthesis of innovative triphenylamine-functionalized organic photosensitizers outperformed the benchmark dye N719 for high-efficiency dye-sensitized solar cells

**DOI:** 10.1038/s41598-022-17041-1

**Published:** 2022-07-28

**Authors:** Safa A. Badawy, Ehab Abdel-Latif, Ahmed A. Fadda, Mohamed R. Elmorsy

**Affiliations:** Department of Chemistry, Faculty of Science, Mansoura University, Mansoura, 35516 Egypt

**Keywords:** Chemistry, Materials science

## Abstract

Herein, we present a thorough photovoltaic investigation of four triphenylamine organic sensitizers with D–π–A configurations and compare their photovoltaic performances to the conventional ruthenium-based sensitizer **N719**. **SFA-5–8** are synthesized and utilized as sensitizers for dye-sensitized solar cell (DSSC) applications. The effects of the donor unit (triphenylamine), π-conjugation bridge (thiophene ring), and various acceptors (phenylacetonitrile and 2-cyanoacetamide derivatives) were investigated. Moreover, this was asserted by profound calculations of HOMO (highest occupied molecular orbital) and LUMO (lowest unoccupied molecular orbital) energy levels, the molecular electrostatic potential (MEP), and natural bond orbital (NBO) that had been studied for the TPA-sensitizers. Theoretical density functional theory (DFT) was performed to study the distribution of electron density between donor and acceptor moieties. The sensitization by the absorption of sensitizers **SFA-5–8** leads to an obvious enhancement in the visible light absorption (300–750 nm) as well as a higher photovoltaic efficiency in the range of (5.53–7.56%). Under optimized conditions, **SFA-7** showed outstanding sensitization of nanocrystalline TiO_2_, resulting in enhancing the visible light absorption and upgrading the power conversion efficiency (PCE) to approximately 7.56% over that reported for the **N719 **(7.29%). Remarkably, **SFA-7** outperformed **N719** by 4% in the total conversion efficiency. Significantly, the superior performance of **SFA-7** could be mainly ascribed to the higher short-circuit photocurrents (*J*sc) in parallel with larger open-circuit voltages (*V*oc) and more importantly, the presence of different anchoring moieties that could enhance the ability to fill the gaps on the surface of the TiO_2_ semiconductor. That could be largely reflected in the overall enhancement in the device efficiency. Moreover, the theoretical electronic and photovoltaic properties of all studied sensitizers have been compared with experimental results. All the 2-cyanoacrylamide derivative sensitizers demonstrated robust photovoltaic performance.

## Introduction

In the unprecedented dilemma of the ever-growing shortage of energy resources and compelling energy demands, dye-sensitizers are considered the holy grail of renewables and the core of dye-sensitized solar cells (DSSCs) because of their availability and pollution-free nature^1^. In 1991, O'Regan and Graztel described a novel type of sensitizer known as DSSCs^2^. They have become promising alternatives to traditional silicon-based photovoltaics because they are cheap, work well, and are easy to make^3^. New attempts have been proposed to present new approaches for the development of dye sensitizers. Most of such dyes are based on metal–organic complexes like ruthenium (Ru) dye sensitizers that are widely used due to their wide optical absorption, high photostability, and energy compatibility with the TiO_2_ layer. This in turn could achieve high DSSC performance associated with various anchoring carboxylate groups via the metal-to-ligand charge transfer (MLCT) processes^4^. Despite the high efficiencies of the sensitized devices with ruthenium-based dyes such as **N3**, **N719**, **black dye**, and **HD-2**, they pose many issues in terms of their high cost, scarcity of metal, and highly purified methods^5–7^. In this regard, metal-free organic sensitizers could be a good choice. There are various types of metal-free sensitizers such as carbazole, phenothiazine, coumarin, thiophene, triphenylamine, and boron dipyrromethene (BODIPY) with classifications D–π–A, D–D–π–A, and D–π–A–A. In comparison to their metal-based counterparts^8^, such as porphyrin dye^9^ and chlorophyll-based dyes^10^, they all have a high molar extinction coefficient, low cost, and ease of purification. Metal-free sensitizers have been widely recognized to have a complementary absorption profile with a high molar extinction coefficient and higher efficiencies^11,12^. Amongst donor units, triphenylamine, phenothiazine, carbazole, and coumarin have been commonly utilized as promising metal-free sensitizers^13^. In this context, TPA sensitizers are characterized by their higher stability, electron-donating capacity, and aggregation resistance, which render them suitable candidates for DSSC applications^14^.

**Figure 1:**
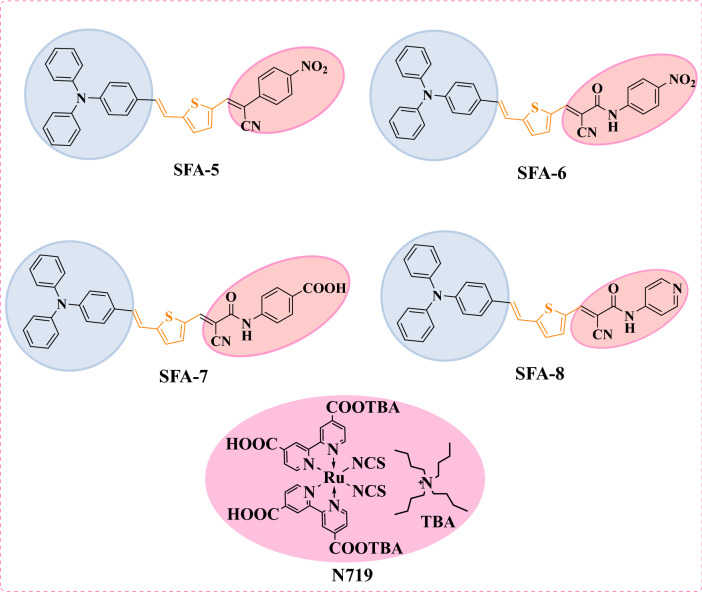
Molecular structures of sensitizers **SFA-5–8 and N719.**

Generally, TPA-based sensitizers can reduce aggregation and allow the interfacial electron injection of excited dye molecules into the TiO_2_ conduction band. Furthermore, TPA compounds can inhibit charge carriers’ recombination of the redox couple (${\text{I}}_{3}^{ -}/{\text{I}}^{-}$) due to their propeller-shaped molecular structure^15^. Such remarkable properties of organic sensitizers are directly related to structure variations, small sizes, and, most importantly, photovoltaic properties. As previously stated, our research team was interested in developing and introducing new co-sensitizers based on D–π–A cyanoacetanilide compounds with multimolecular structures and a variety of acceptor and donor moieties. Furthermore, an ideal sanitizer should have specific functional groups called anchoring groups to enable strong binding between the dye and the surface of the semiconductor oxide^16^. Historically, the most frequently used anchors in DSSCs are carboxylic acid and cyanoacrylic acid groups^17–23^. The anchoring groups link with the TiO_2_ surface to enable the injection of the excited electron into the CB of the TiO_2_^24^. Due to the exponential growth of DSSC research in recent years, many new anchors have been made and tested. This has greatly increased the number of materials available and made it easier to understand DSSCs. Against this background, we present the design, synthesis, characterization, and photovoltaic performance of four innovative sensitizers stated as **SFA-5–8,** containing a triphenylamine moiety as a donor group attached to π-bridge (thiophene moiety) linked to different 2-cyano-acrylamide acceptors and phenyl acetonitrile units. Such D–π–A **SFA-5–8** models have been schematically represented in Fig. 1. In addition, 2-cyano-acrylamide and phenyl acetonitrile units containing electron-withdrawing groups (EWGs) as CN, CO, NO_2_, and amide (CONH) are of great importance in organic synthesis due to their strong accepting ability^25,26^. Hence, 2-cyano-acrylamide acceptor and phenyl acetonitrile units were chosen because they possess the good electron-accepting ability and, consequently, facilitate effective interaction with TiO_2_^27^. In this connection, the development of novel acceptors/anchors for dye-sensitizers is a very crucial task to enhance the performance of DSSCs. Accordingly, higher efficiencies of approximately 5.53–7.56% have been achieved by our synthesized **SFA-5–8** sensitizers compared to that reported for the standard **N719** dye (7.25%).

## Experimental

### Materials and methods

The detergents, chemicals, and solvents necessary for the chemical reactions and synthetic procedures were purchased from Sigma-Aldrich, TCI America, and Alfa Aesar, respectively, and utilized exactly as supplied. The measured melting points (uncorrected) are just in degrees Celsius, employing a Gallenkamp electric melting point instrument. A Thermo Scientific Nicolet iS10 FTIR spectrometer was used for identifying the IR spectra (KBr). Nuclear magnetic resonance (NMR) spectra were obtained on a Bruker NMR spectrometer using DMSO-*d*6 as a solvent at 400 MHz (^1^H NMR) and 100 MHz (^13^C NMR) with an internal standard (TMS), and chemical shifts are given as δ/ppm. UV–Visible spectra were measured by using the high-performance double beam spectrophotometer (T80 series). A Thermo DSQ II spectrometer was used to record mass analyses. The Perkin Elmer 2400 analyzer was used to collect data for the elemental analysis. Finally, in the accompanying information file, all instruments and DSSCs fabrications were thoroughly discussed.

#### Synthesis of 5-(4-(diphenylamino)styryl)thiophene-2-carbaldehyde (6)

Thiophene-2-carbaldehyde compound **6** has been synthesized through two reactions, firstly phosphonium salt and 2-formylthiophene react via Wittig reaction under alkaline conditions (*t*-BuOK), At 50 mL two-neck RB flask, 19 mL of freshly distilled POCl_3_ was added dropwise to the (20 mL) of a stirred solution of dry DMF at 0 °C under argon atmosphere until colored Vilsmeier salt completely precipitates. Then a solution of **5** (0.54 g of *N*,*N*-diphenyl-4-(2-(thiophene-2-yl)vinyl)aniline dissolved in 10 mL DMF) was added to the reaction mixture drop by drop with continuous stirring for 1 h. The temperature was increased to 120 °C for 2 h, then the mixture was stirred overnight at room temperature. After completion of the reaction, the mixture was poured into 100 mL of ice-cold water and the pH was adjusted to alkaline by adding saturated sodium acetate solution. The solid product was collected by filtration and synthesized as a previously reported procedure^28^. The targeted **SFA-5–8** sensitizers were synthesized via a Knoevenagel reaction that was applied on methylene compounds [such as 2-(4-nitrophenyl)acetonitrile] and different cyanoacetamide derivatives **7a–c**^29,30^.

#### 3-(5-(4-(Diphenylamino)styryl)thiophene-2-yl)-2-(4-nitrophenyl)acrylonitrile (SFA-5)

2-(4-Nitrophenyl)acetonitrile (**7a**) (0.33 g, 2 mmol) and 5-(4-(diphenylamino)styryl)-thiophene-2-carbaldehyde **(6)** (0.76 g, 2 mmol) were in 50 mL methanol in a round-bottomed flask. To this reaction mixture, drops of 1,8-diazabicyclo[5.4.0]undec-7-ene (DPU) were added, then the contents of the flask were heated for 5 h at 80 °C. The reaction was cooled at room temperature and the mixture was poured into ice water and neutralized with diluted 1 M HCl to yield reddish-brown compound. Yield = 75%, m.p. = 196–198 °C. IR (KBr): *ν*_*max*_ 2922 and 2854 (C–H), 2209 (C≡N), 1612 cm^−1^ (C=C). ^1^H NMR (DMSO*-d*_*6*_): *δ* 6.97 (d, *J* = 12.00 Hz, 2H, C=CH), 7.20–7.29 (m, 7H, Ar–H), 7.45 (t, *J* = 6.00 Hz, 5H, Ar–H), 7.56–7.64 (m, 6H, Ar–H and thiophene-H), 7.94 (s, 1H, C = CH), 8.15 ppm (d, *J* = 8.00 Hz, 2H, Ar–H). ^13^C NMR (DMSO-*d*_6_): *δ* 111.32, 119.61, 120.75, 121.49, 126.43 (2C), 126.74, 126.81, 127.09, 127.49, 127.78 (2C), 128.73 (4C), 133.35 (4C), 134.51 (2C), 134.81 (2C), 139.90, 146.49 (2C), 147.46, 148.60, 155.13 (2C), 156.31, 160.93 ppm. Analysis calcd. for C_33_H_23_N_3_O_2_S (525.15): C, 75.41; H, 4.41; N, 7.99%. Found: C, 75.21; H, 4.50; N, 7.87%.

#### 2-Cyano-3-(5-(4-(diphenylamino)styryl)thiophen-2-yl)-N-(4 nitrophenyl)acrylamide (SFA-6)

5-(4-(Diphenylamino)styryl)thiophene-2-carbaldehyde **(6)** (0.76 g, 2 mmol) and 2-cyano-N-(4-nitrophenyl)acetamide (0.41 g, 2 mmol) were dissolved in methanol (15 mL). Acetic acid (0.20 mL) was added to the mixture, reflux was continued for 18 h and then cooled to room temperature. The solid that obtained was purified using silica gel column chromatography to obtain dark brown solid compound. Yield = 88%, m.p. = 190–192 °C. IR (KBr): *ν*_*max*_ 3330 (N–H), 2222 (C≡N), 1675 cm^−1^ (C = O). ^1^H NMR (DMSO*-d*_*6*_): *δ* 6.96 (d, *J* = 12.00 Hz, 1H, C = CH), 7.74 (d, *J* = 12.00 Hz, 1H, C = CH), 7.90 (t, *J* = 6.00 Hz, 3H, Ar–H), 8.05–8.14 (m, 11H, Ar–H), 8.21 (d, *J* = 4.00 Hz, 1H, thiophene-H), 8.24 (d, *J* = 8.00 Hz, 2H, Ar–H), 8.46 (d, *J* = 4.00 Hz, 1H, thiophene-H), 8.49 (d, *J* = 8.00 Hz, 2H, Ar–H), 8.93 (s, 1H, C=CH) ppm. ^13^C NMR (DMSO-*d*_6_): *δ* 112.05, 120.23 (2C), 121.11, 122.35, 126.05, 126.36 (4C), 126.43 (2C), 126.71, 127.10 (4C), 127.39, 130.03, 134.13, 134.42 (3C), 140.03, 147.08 (2C), 148.20, 149.28, 150.29 (2C), 154.74, 155.97, 156.04, 160.54 ppm. Analysis calcd. for C_34_H_24_N_4_O_3_S (568.16): C, 71.81; H, 4.25; N, 9.85%. Found: C, 71.76; H, 4.18; N, 9.95%.

#### 4-(2-Cyano-3-(5-(4-(diphenylamino)styryl)thiophen-2-yl)acrylamido)benzoic acid (SFA-7)

To 50 mL ethanol, 0.76 g (2 mmol) of 5-(4-(diphenylamino)styryl)thiophene-2-carbaldehyde (**6**) and 4-(2-cyanoacetamido)benzoic acid (**7c**) (0.40 g, 2 mmol) and three drops of piperidine were added. The solution was refluxed for 3 h. After cooling to room temperature, the mixture was poured into ice water. The brown precipitate was filtered and washed with water. After drying, the precipitate was purified by recrystallization from a mixture of e ethanol and drops of acetic acid. Yield = 69%, m.p. = 172–174 °C. IR (KBr): *ν*_*max*_ 3334 (N–H), 2220 (C≡N), 1722 (C=O), 1661 (C=O) cm^−1^. ^1^H NMR (DMSO*-d*_*6*_): *δ* 6.69 (d, *J* = 12.00 Hz, 2H, C=CH), 7.73–7.77 (m, 9H, Ar–H), 7.93 (t, *J* = 8.00 Hz, 2H, Ar–H), 8.07 (t, *J* = 8.00 Hz, 1H, Ar–H), 8.14 (d, *J* = 4.00 Hz, 4H, Ar–H), 8.18 (d, *J* = 4.00 Hz, 1H, thiophene-H), 8.27 (d*, J* = 4.00 Hz, 1H, thiophene-H), 8.43 (s, 1H, C=CH), 8.52 (d, *J* = 8.00 Hz, 2H, Ar–H), 13.67 (s, 1H, COOH). ^13^C NMR (DMSO-*d*_6_): *δ* 112.16 (2C), 117.64, 121.09 (2C), 122.35 (4C), 126.06 (2C), 126.73 (2C), 127.40 (2C), 130.80 (2C), 134.15 (2C), 134.44 (2C), 140.24, 148.20 (2C), 149.29, 150.29 (3C), 150.77 (2C), 154.81 (2C), 155.97, 156.36, 169.00 ppm. Analysis calcd. for C_35_H_25_N_3_O_3_S (567.16): C, 74.06; H, 4.44; N, 7.40%. Found: C, 74.26; H, 4.52; N, 7.53%.

#### 2-Cyano-3-(5-(4-(diphenylamino)styryl)thiophen-2-yl)-N-(pyridin-4-yl)acrylamide (SFA-8)

To a suspension of **6** (0.76 g, 2 mmol) and 2-cyano-*N*-(pyridin-4-yl)acetamide (**7d**) (0.32 g, 2 mmol) in 15 mL ethanol, 0.2 mL piperidine has been added. The reaction mixture was refluxed for 10 h and the pure sensitizer **SFA-8** was separated by filtration after cooling the solution to room temperature for obtaining dark brown crystals. Yield = 80%, m.p. = 184–186 °C. IR (KBr): *ν*_*max*_ 3324 (N–H), 2219 (C≡N), 1679 (C=O) cm^−1^. ^1^H NMR (DMSO-*d*_*6*_): *δ* 6.95 (d, *J* = 12.00 Hz, 1H, C=CH), 7.74 (d, *J* = 12, 1H, C = CH), 7.90 (t, *J* = 6.00 Hz, 2H, Ar–H), 8.07 (t, *J* = 8.00 Hz, 4H, Ar–H), 8.11–8.14 (m, 5 H, Ar–H and thiophene-H), 8.20 (d, *J* = 8.00 Hz, 2H, Ar–H), 8.24 (d, *J* = 8.00 Hz, 2H, Ar–H), 8.40 (s, 1H, C=CH), 8.46 (d, *J* = 4.00 Hz, 1H, thiophene-H), 8.49 (d*, J* = 4.00 Hz, 2H, pyridine-H), 8.92 (d, *J* = 4.00 Hz, 2H, pyridine-H). ^13^C NMR (DMSO-*d*_6_): *δ* 110.93 (2C), 119.35, 121.15, 126.05 (2C), 126.66, 127.19 (4C), 127.40 (2C), 128.05 (4C), 128.09, 128.16, 128.35, 128.47, 132.97, 134.00, 134.09 (2C), 143.30, 146.12, 148.23 (2C), 154.76, 155.14, 155.95, 168.04 ppm. Analysis calcd. C_33_H_24_N_4_O_S_ (524.17): C, 75.55; H, 4.61; N, 10.68%. Found: C, 75.77, H, 4.51, N, 10.52%.

### Results and discussion

#### Synthesis and structure characterization

**Figure 2:**
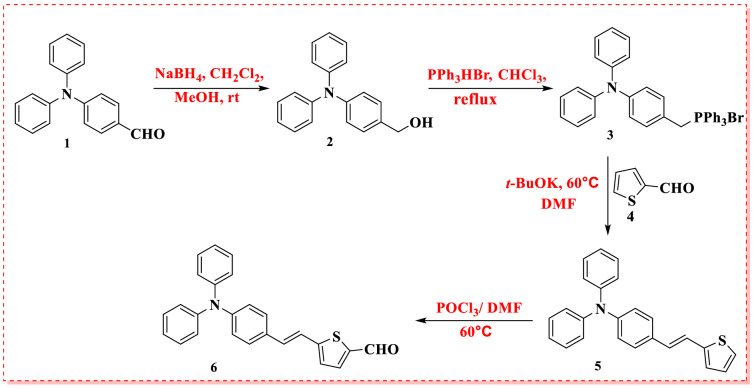
Synthesis of 5-(4-(diphenylamino) styryl)thiophene-2-carbaldehyde (**6**).

**Figure 3:**
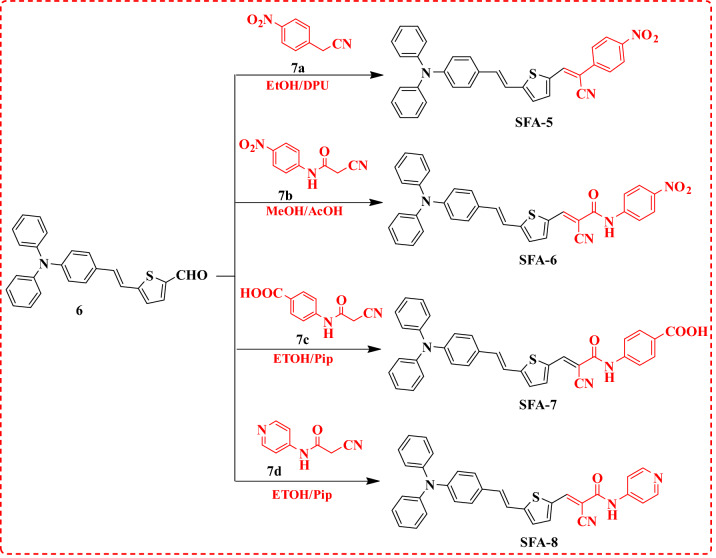
Synthetic routes of **SFA-5–8** sensitizers**.**

The synthetic pathways of four new triphenylamine-based organic compounds (**SFA-5–8**) are depicted in Figs. 2 and 3. Figure 2 shows the synthetic pathway of 5-(4-(diphenylamino) styryl) thiophene-2-carbaldehyde (**6**), including the reaction of 4-((bromotriphenyl-λ^5^-phosphanyl)methyl)-*N*,*N*-diphenylaniline (**3**) with 2-formylthiophene (**4**) under Wittig reaction conditions, obtaining the corresponding thiophene bridge (**5**). The intermediate **5** was then formulated using the standard Vilsmeier-Hack reaction protocol to yield 5-(4-(diphenylamino) styryl)thiophene-2-carbaldehyde (**6**) with a good overall yield (Fig. 2). The observed melting point and spectroscopic characterization of triphenyl amine-based compounds 5 and 6 were well-matched with previous studies^31^.

Afterward, 2-cyanoacetamide derivatives **7b–d** were synthesized in a high yield via the refluxing of various aromatic amines such as 4-nitro aniline, 4-aminobenzoic acid, and 4-aminopyridine with 1-cyanoacetyl-3,5-dimethylpyrazole in dioxane as a solvent, as previously stated in the literature^29,30^. Finally, the targeted final products **SFA-5–8** were formed in a high yield *thru* Knoevenagel condensation of 5-(4-(diphenylamino) styryl)thiophene-2-carbaldehyde (**6**) with 2-(4-nitrophenyl)acetonitrile **(7a)** and three 2-cyanoacetamide derivatives **7b, 7c** and **7d** as shown in Fig. 3. The end products **SFA-5–8**, as well as their intermediates, were extensively purified using column chromatography in addition to the recrystallization process. The structures of the newly synthesized sensitizers and their intermediates were confirmed by various spectroscopic techniques. According to their spectral analysis and elemental investigation, the molecular structure of **SFA-5–8** was determined. The IR spectrum of compound **SFA-5** revealed characteristic absorption bands of groups, C–H aliphatic at 2922 and 2854 cm^−1^, and cyano group (C≡N) at 2209 cm^−1^_._ Moreover, the stretching vibration ban of vinyl groups (C=C) appeared at 1612 cm^−1^. Its corresponding ^1^H NMR spectrum exhibited a doublet signal at *δ* 6.97 ppm that could be attributed to the two protons of the vinylic group with (*J* = 12.00 Hz), singlet for the olefinic proton at *δ* 7.94 ppm. The aromatic and thiophene protons resonate as multiplet, triplet, and doublet signals at *(δ* 7.20–8.15 ppm). Additionally, the IR spectrum of compound **SFA-6** exhibited stretching vibration bands at 3330 cm^−1^ and 2222 cm^−1^ due to the N–H and C≡N moieties, respectively. Moreover, a strong band at 1675 cm^−1^ has been detected that could be assigned to the C=O group. The visual inspection of the corresponding ^1^HNMR spectral data revealed a singlet peak at 8.93 ppm corresponding vinylic proton. Further, The IR spectrum of **SFA-7** indicated a vibration band of (C≡N) group at 2220 cm^−1^ as well as a strong vibration band at 1722 cm^−1^ attributed to C=O (COOH). Meanwhile, the ^1^H NMR spectrum of **SFA-7** showed a singlet signal at 13.67 ppm associated with the carboxylic group proton. On the other hand, the ^13^C NMR spectra revealed a characteristic signal at *δ *169.00 ppm which could be linked to the carbon of the COOH group. Finally, the IR spectrum of **SFA-8** reported absorption bands at 2219 cm^−1^ and 1679 cm^−1^ that can be properly assigned to the stretching vibration modes of (C≡N) and (C = O) groups, respectively. Additionally, the ^13^CNMR spectra revealed a characteristic signal at *δ *168.04 ppm, ascribable to the carbon of the carbonyl group.

#### Optical properties

The absorption spectra of all synthesize **SFA-5–8 sensitizers** have been recorded in tetrahydrofuran (THF) solution. Their corresponding data are summarized in Table 1.

**Table 1:** UV–Vis absorption spectra of the synthesized **SFA-5–8** sensitizers.

Sensitizer	λ_Max_ (nm)	Ɛ (10^4^ M^−1^ cm^−1^)	λ_Onset_/nm	*Experimental* *E*_0–0_ (eV)
SFA-5	256, 354, 468	3.20, 0.84, 1.77	541	2.29
SFA-6	254, 356, 481	3.81, 0.97, 2.32	580	2.13
SFA-7	253, 345, 478	3.78, 1.88, 2.22	562	2.20
SFA-8	255, 342, 474	3.11, 1.02, 1.95	554	2.23

**Figure 4:**
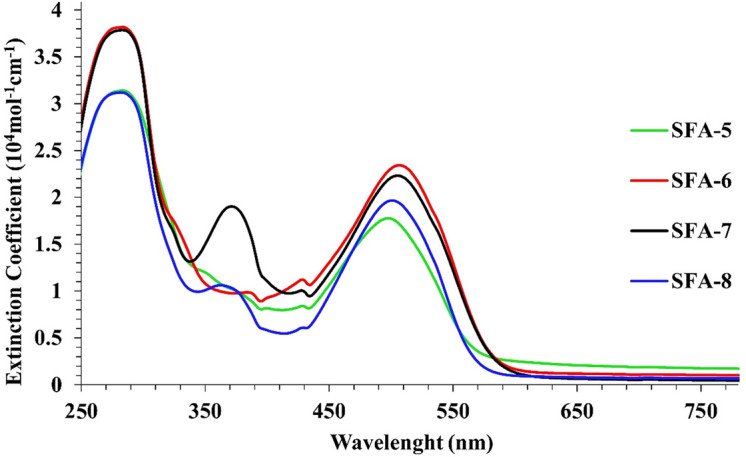
UV–Vis absorption of sensitizers **SFA-5–8** measured in THF (2 × 10^−5^ M).

It is generally recognized that organic sensitizers show two distinct principal absorption bands at shorter (250–400 nm) and longer (420–600 nm) wavelengths. Note that the noticeable bands at shorter wavelengths could be assigned to the π–π* transitions. As for the strong absorption peak observed in the visible spectrum region (420–600 nm), it could be mainly related to the intramolecular charge transfer (ICT) from the triphenylamine (donor) to the acceptor moieties^32^. It is important to note that sensitizers **(SFA-5–8)** demonstrate intense absorption peaks in the visible region, specifically for **SFA-5** that can be linked to the ICT from triphenylamine donor to the CN, and NO_2_ acceptors parts of the nitrophenyl acetonitrile unit. That has been changed to **SFA-6–8 which,** showed transitions to the CN, CO, COOH, NO_2_, and pyridine over 2-cyanacetamide derivatives. Notably, it has been observed that the absorption peak maxima (*λ*_max_ = 400–500 nm) in THF solution for all synthesized sensitizers followed the order of **SFA-5** < **SFA-8** < **SFA-7** < **SFA-6**. That could be linked to the addition of the π-bridge thiophene group to the **SFA-5–8** sensitizers that significantly contributed to energy delocalization and larger polarizability, thus enhancing the visible light absorption^32^. Moreover, the estimated energy gap (*E*_0–0_), which was calculated from the beginning of the UV–visible absorption spectrum^33^, has been minimized with the presence of the π-bridge linkage, which promoted visible light absorption. Those values followed the order of **SFA-6 < SFA-7 < SFA-8 < SFA-5.** Importantly, the λ_max_ of **SFA-5–8** in the visible region appears at 468, 481, 478, and 474 nm, respectively. As expected, electronic density variations between the donating electron and the withdrawing part could imply a bathochromic shift in the internal charge transfer (ICT) band^33^, thus reporting molar extinction coefficients (**ε**) of approximately 1.77 × 10^4^ mol^−1^ cm^−1^, 2.32 × 10^4^ mol^−1^ cm^−1^, 2.22 × 10^4^ mol^−1^ cm^−1^, and 1.95 × 10^4^ mol^−1^ cm^−1^ for **SFA-5–8**, respectively, associated with their lowest energy bands. Those mentioned values are significantly higher than those reported for the Ru **N719** dyes, indicating better light-harvesting ability^33^. For comparison, the UV–Vis absorption spectrum of **N719** has been reported in Fig. S1 in the “Supplementary file”. Further insights showed that **SFA-6–8** was more red-shifted in contrast to its corresponding mono-anchoring **SFA-5** dye. This can be attributed to the extension of conjugation length within the synthesized SFA-6-8 molecules, as well as the delocalization across the entire molecule, which could be caused by the addition of an additional anchoring moiety over 2-cyanoacetamide derivatives^34^. In practical, **SFA-6** comprises 4-nitrocyanoacetamide bearing CN, NO_2_, and CO substitutions that are mainly responsible for the bathochromic shift with the highest ε value of approximately 2.32 × 10^4^ mol^−1^ cm^−1^ amongst all studied dyes, as displayed in Fig. 4. That could be ascribed to the presence of the strongly withdrawing (–NO_2_) moiety in their anchoring function. Furthermore, it could be deduced that **SFA-7** has more bathochromic shift by around 4 nm, compared to its **SFA-8** counterpart, which is associated with its higher degree of conjugation.

**Figure 5:**
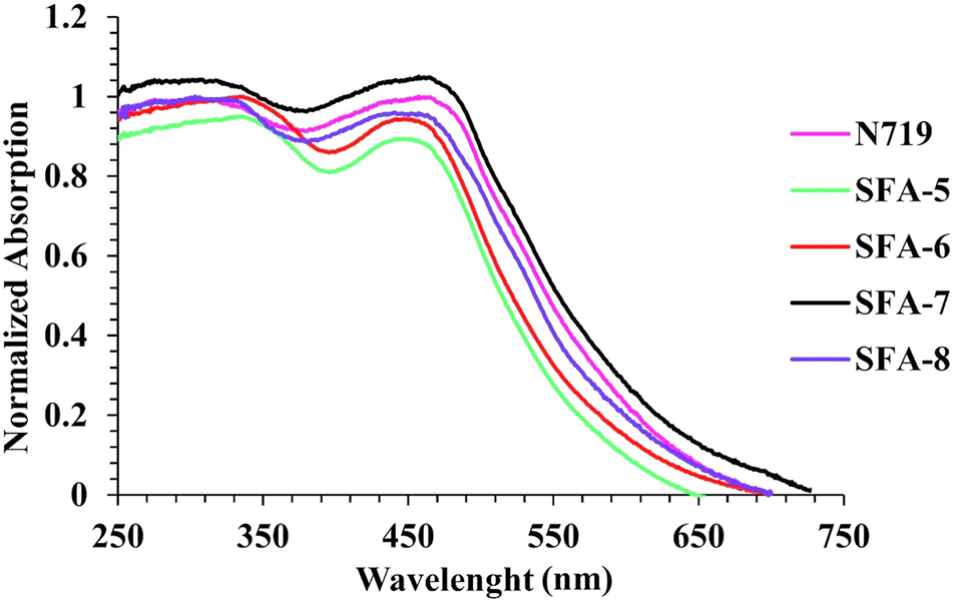
Absorption spectra of **SFA-5–8** adsorbed on nonporous TiO_2_.

Figure 5 shows the absorption spectra of triphenylamine sensitizers **SFA-5–8** anchored on the TiO_2_ surface. As could be observed, all **SFA-5–8** sensitizers showed broadening and blue shifting in the absorption spectra compared to their corresponding spectra recorded in the solution spectrum (250–550 nm). This hypochromic shift is directly related to *H*-aggregation and strong interaction between the anchoring groups of sensitizers and the semiconductor photoelectrode, which is highly desirable for achieving efficient light-harvesting and promoting the overall efficiency of sensitizers^35^. In particular, it should be emphasized that the observed spectral broadening for **SFA-7** could be extensively beneficial for enhancing the visible light-harvesting ability and enhancing the short circuit current (*Jsc)*. The highest absorbance values exhibited by the **SFA-7** sensitizer compared to other sensitizers can be mainly ascribed to the deprotonating of carboxylic acid or the formation of H-aggregates on the TiO_2_ surface, which lead to lowering the π* energy level of its 4-carboxylcyanacetamide group and thereby broadening its absorption spectrum. That in turn could confirm the good ability of the 4-carboxylcyanoacetamide of the **SFA-7** sensitizer to harvest more photons compared to that of the **N719** counterpart. Contrarily, **SFA-5** showed the weakest absorbance characteristics compared to other 2-cyanoacetamide derivatives of **SFA-6–8** sensitizers, thus confirming the direct effect of anchoring and acceptor groups on the absorbance enhancement. Thereby, we believe that including extra anchoring groups in the design of sensitizers could in turn promote the dye adsorption on the TiO_2_ surface, narrow the spectrum, and most importantly facilitate the electron flow from the excited dyes to the TiO_2_ conduction band^36^.

#### Theoretical calculations

**Figure 6:**
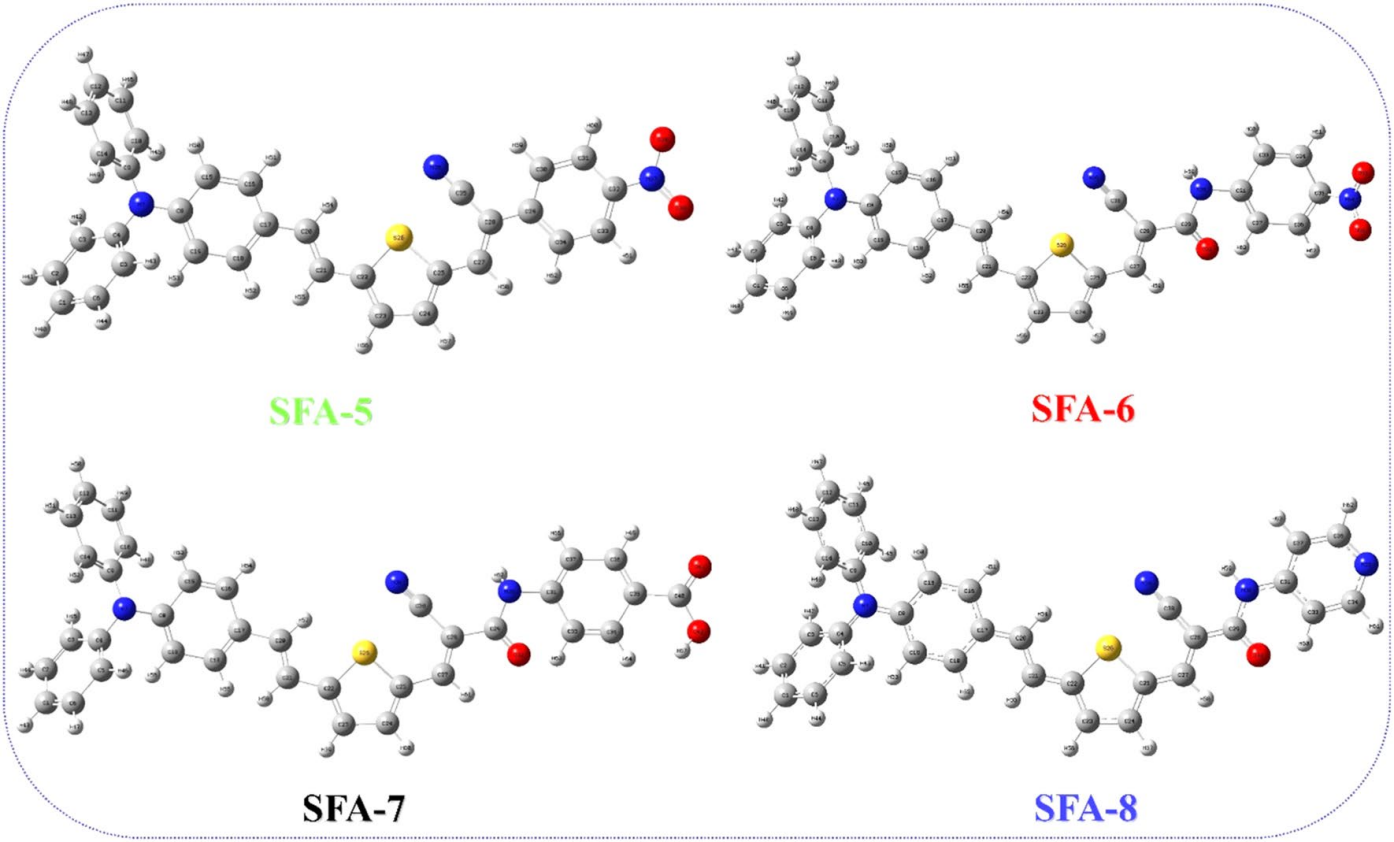
Illustration of the optimized **SFA-5–8** dye sensitizer structures.

Computational studies for **SAF-5–8** dyes were established to unravel how the characteristic π-spacer and different anchoring/acceptors moiety *thru* phenylacetonitrile and cyanoacetamide derivative sensitizers influence the geometry of the targeted dyes and their DSSC photovoltaic efficiency. The details of the calculations are based on Gaussian 09 software^37^ via B3LYP/6-311 g (d, p) sets^38^. As demonstrated in Fig. 6, the optimized geometrical structures of the **SFA-5–8** have been reported. Moreover, their corresponding dihedral angles and bond lengths have been summarized in Table 2. All **SFA-5–8** dyes show a propeller starburst arrangement and (**D–π**) dihedral angles of about (-179, 179, -177, and 179) with π-bridges that could be of great importance for achieving efficient charge transfer.

**Table 2:** Selected dihedral angles and bond lengths of the **SFA-5–8** sensitizers**.**

Sensitizers	Dihedral angle (°)			Bond length (Å)		
	D–π (°)	π–π (°)	π–A (°)	D–π (Å)	π–π (Å)	π–A (Å)
SFA-5	− 179	0	179	1.50	1.34	1.50
SFA-6	179	179	179	1.50	1.34	1.34
SFA-7	− 177	174	180	1.42	1.34	1.32
SFA-8	179	− 179	179	1.50	1.36	1.44

Note that inserting the thiophene moiety is advantageous for increasing the conjugation degree of the **SFA-5–8** sensitizers. Meanwhile, DFT calculations showed that **SFA-5–8** structures possessed higher dihedral angles that are close to 180° or equal to zero. Such values could reflect better coplanarity configurations and favorable conjugation between the thiophene-π-spacer and the acceptor moiety^38^. Indeed, those findings could infer the highest λ_max_ values of the **SAF-5–8** structures which is a good indicator of the fast electron transfer to the semiconductor surface, resulting in boosting the overall performance. For further insights, Table 2 indicated the bond lengths of **SFA-5** as **D–π** (1.50 Å), **π–π** (1.34 Å), **π–A** (1.50 Å), **SFA-6** as **D–π** (1.50 Å), **π–π** (1.34 Å), **π–A** (1.34 Å), **SFA-7** as **D–π** (1.42 Å), **π–π** (1.34 Å), **π–A** (1.32 Å) and **SFA-8** as **D–π** (1.50 Å), **π–π** (1.36 Å), **π–A** (1.44 Å). Because of the steric hindrance between the donor (D), π-bridge, and acceptors units, we can theoretically determine the co-planarity of the compounds through the dihedral angle calculation. Therefore, designated dye sensitizers have a significant potential to avoid unfavorable dye aggregation (**π–π**). It is well documented that the dye aggregation mainly contributes to the self-quenching of excitation and hence lowers the efficiency of the electron injection. Therefore, even the dye molecules’ structural geometry gave a valuable foresight about the perceptions of their improved photovoltaic performances as reference^38^. All of these computed findings revealed a strong substantial conjugation impact that could strongly stimulate the electron transfer from the donating TPA moieties to the different electron acceptors over **SFA-5–8** structures.

#### Natural bond orbital analysis

To further elucidate the origin of the intramolecular interactions and give deep insights into the intramolecular electron transfer processes within the synthesized **SFA-5–8** sensitizers, NBO analysis could be of crucial importance. In this context, NBO analysis could study the stabilizing interactions between the filled (donor) and empty (acceptor) orbitals as well as the destabilizing interactions between the filled orbitals^39^. By using the second-order perturbation approach, the hyper conjugative interaction energy was estimated from a donor (***i***), an acceptor (***j***), and the stabilization energy ***E***^***(2)***^ associated with the delocalization ***j, i*** could be estimated as the following equation:

1 $${{\varvec{E}}}^{(2)}={\boldsymbol{\Delta }{\varvec{E}}}_{{\varvec{i}},{\varvec{j}}}={{\varvec{q}}}_{{\varvec{i}}}\frac{{{\varvec{F}}({\varvec{i}},{\varvec{j}})}^{2}}{{{\varvec{\varepsilon}}}_{{\varvec{i}}}-{{\varvec{\varepsilon}}}_{{\varvec{j}}}}$$

where ***qi*** represents the donor orbital occupancy, ${{\varvec{\varepsilon}}}_{{\varvec{i}}}\boldsymbol{ }\text{and}\boldsymbol{ }{{\varvec{\varepsilon}}}_{{\varvec{j}}}$ the diagonal elements and ***F(i, j)*** is the off-diagonal. In NBO studies, strong intermolecular hyper conjugative interactions were analyzed by the second-order perturbation theory of the Fock matrix. For **SFA-5–8** sensitizers, the higher energy values of ***E***^***(2)***^ hyper conjugative interactions, the more intensive interaction between an electron donor (TPA moiety) and electron acceptors (NO_2_, COOH, CN, and pyridyl ring), means the greater ability to donate tendency from electron donor parts to electron acceptors. The strong stabilization energy for **SFA-5** sensitizer is from π* (C16–C17) → π* (C20–C21), π*((C8–C15) → π*(C16–C17), π (C31–C32) → π* (N37–O39) have the highest ***E***^***(2)***^ values 231.78, 111.85, 68.82 kcal/mol, respectively. This electron transition represents electron transfer across the sensitizer from triphenylamine (donor) to thiophene (system) ring to phenyl acetonitrile (acceptor), indicating effective charge transfer^40^. Similarly, in the case of **SFA-6,** important interaction π*(C24–C25 → π*(C22–C23), π*(C35–C36) → π*(C31–C37), and π*(C29–O32) → π*(C27–C28) have the highest ***E***^***(2)***^ values of 85.04, 36.74 and 30.43 kcal/mol, respectively. For **SFA-7**, there occurs a strong intramolecular hyper conjugative interaction from D–π–A, represented in π*(C10–C11) → π*(C12–C13), π*(C10–C11) → π*(C9–C14), π*(C40–O41) → π*(C35–C36) and π*(C22–C32) → π*(C20–C21) have the highest **E**^**(2)**^ values of 68.43, 50.03, 29.89 and 26.21 respectively. **SFA-8** transition π*(C24–C25 → π*(C22–C23) which leads to strong delocalization, π*(C35–C36) → π*(C31–C37) and π*(C29–O32) → π*(C27–C28) showed the highest ***E***^***(2)***^ values 90.04, 60.78 and 50.98 kcal/mol, respectively.

Quantum chemical parameter SFA-5-8.

The chemical reactivity of the **SFA-5–8** sensitizers could be analyzed by utilizing the quantum analysis by calculating several significant factors; including the bandgap energy *(E*_*0-0*_), ionization energy *(IP),* electron affinity *(EA),* hardness *(η)*, and softness (s), respectively. With respect Koopmans’ hypothesis framework^41^, the corresponding data are displayed in Table 3.

**Table 3:** Quantum chemical parameters of the **SFA-5–8** synthesized sensitizers.

Dye	HOMO	LUMO	E_0-0_	IP	EA	(s)	η	∆G_inject_ (eV)	∆G_reg_ (eV)	∆G_rec_ (eV)
SFA-5	**− **5.96	**− **3.69	2.27	5.96	3.69	0.88	1.13	**− **0.51	0.76	1.76
SFA-6	**− **5.70	**− **3.50	2.20	5.70	3.50	0.90	1.10	**− **0.70	0.50	1.50
SFA-7	**− **5.54	**− **3.44	2.10	5.54	3.44	0.95	1.05	**− **0.76	0.34	1.34
SFA-8	**− **5.65	**− **3.47	2.18	5.65	3.47	0.91	1.09	**− **0.73	0.45	1.45

2 $$IP=-{E}_{HOMO}$$

3 $$EA=-{E}_{LUMO}$$

4 $$\eta =\left(\frac{{E}_{LUMO}-{E}_{HOMO}}{2}\right)$$

The HOMO–LUMO energy gaps of the **SFA-5–8** decreased in a sequence of **SFA-7 < SFA-8 < SFA-6 < SFA-5.** The smallest *E*_*0–0*_ of the **SFA-7** structure could reveal its highest stability and confirm its reactive configuration amongst all studied sensitizers, thus allowing for more dominant excitation between the HOMO and LUMO of the **SFA-7** dye molecule. Accordingly, 4**-**carboxycyanoacetamide **SFA-7** sensitizer should be chemically more reactive than other **SFA-5**, **SFA-6**, and **SFA-8** dyes. On the other hand, hardness ***(η)*** and softness **(s**) are very useful parameters that could reflect the system's reactivity. On one hand, hardness could assess the maximal electromotive force between donors and electron acceptors within the same molecule. In this regard, accurate determination of energy gaps can be used to classify a substance as either hard or soft. For instance, a high HOMO–LUMO gap means a hard species, whereas a low HOMO–LUMO gap indicates a soft moiety^41^. Importantly, the global hardness of a pure substance was linked to its stability and reactivity. The reactivity is inversely linked to global hardness, while the stability is closely linked to it^41^. Further insights, the global hardness for **SFA-5–8** sensitizers was reported to be ***η*** = 1.13 > 1.10 > 1.05 > 1.09 eV, respectively. The lower hardness (η) value could be appropriate for allowing an effective charge transfer within the dye molecules. Thereby, **SFA-7** could show efficient charge transfer features when compared to its corresponding sensitizes **(SFA-5–8)**. Further, the key photovoltaic parameters such as ∆G_inj._, ∆G_reg._, and ∆G_rec._ were estimated as shown in Eqs. (5–9)^42^ and their related data were summarized in Table 3.

5 $${\text{Where}; E}_{OX}^{dye}=-HOMO$$

6 $${E}_{OX}^{{dye}^{*}}= {E}_{OX}^{dye}-{E}_{0-0}$$

7 $$\Delta {\text{G}}_{\text{inj}}\left(\text{eV}\right)={E}_{OX}^{{dye}^{*}}-{E}_{CB}$$

8 $${\Delta {\text{G}}_{\text{reg}}\left(\text{eV}\right)=\text{E}}_{\text{OX}}^{\text{dye}}-{E}_{redox}$$

9 $${\Delta {\text{G}}_{\text{rec}}\left(\text{eV}\right)=\text{E}}_{\text{OX}}^{\text{dye}}-{E}_{CB}$$

ΔG_inj._ for **SFA-5–8 molecules** was calculated by ${E}_{OX}^{dye*}$ and *E*_HOMO_ and it was found to be − 0.51, − 0.70, − 0.76, and − 0.73 eV, respectively. This could forecast an adequate driving force for the excited organic molecules to inject electrons into the conduction band (CB) of theTiO_2_ molecule. That in turn could enhance the *Jsc* values and promote the DSSC efficiency. It could be observed that all driving force values (ΔG_inj_) of the synthesized **SFA-5–8** dyes are negative, which is ideal for hole injection. Most importantly, **SFA-7** had a greater value than the other TPA dyes, confirming that adding one additional acceptor and anchoring moieties (COOH, CN, CO, NH) to the dye molecule could facilitate the charge transfer features, thus better DSSC performance^42^. For comparison, the predictable dye regeneration values are listed in Table 3 in an ascending order as fellow: **SFA-5** (0.76 eV) > **SFA-6** (0.50 eV) > **SFA-8** (0.45 eV) > **SFA-7** (0.34 eV). As reported in Table 3, **SFA-7** has the smallest ΔG_reg._ value of ~ 0.10 eV, demonstrating the maximum dye regeneration capability. Those who reported ΔG_rec._ values have been decreased as the following **SFA-7** (1.34 eV) < **SFA-8** (1.45 eV) < **SFA-6** (1.50 eV) < **SFA-5** (1.76 eV). When **SFA6–8** sensitizers were injected, three fractions carrying the 2-cyanoacrylamide derivatives with different acceptors had been proposed which could defeat the charge recombination to a specific limit. In this context, **SFA-7** has reported the lowest charge recombination value (ΔG_rec_) of about 1.34 eV in comparison with other structures in the same class. Accordingly, based on the calculated outcomes of the aforementioned factors ΔG_inj_, ΔG_reg_, and ΔG_rec_, **SFA-7** could confirm its superiority as a high efficient dye for DSSCs applications. Moreover, those previous findings could suggest that phenyl acetonitrile and 2-cyanonacetamide of **SFA-5–8** compounds might be also viable dyes as good choices for DSSC.

#### Electrochemical properties

**Figure 7:**
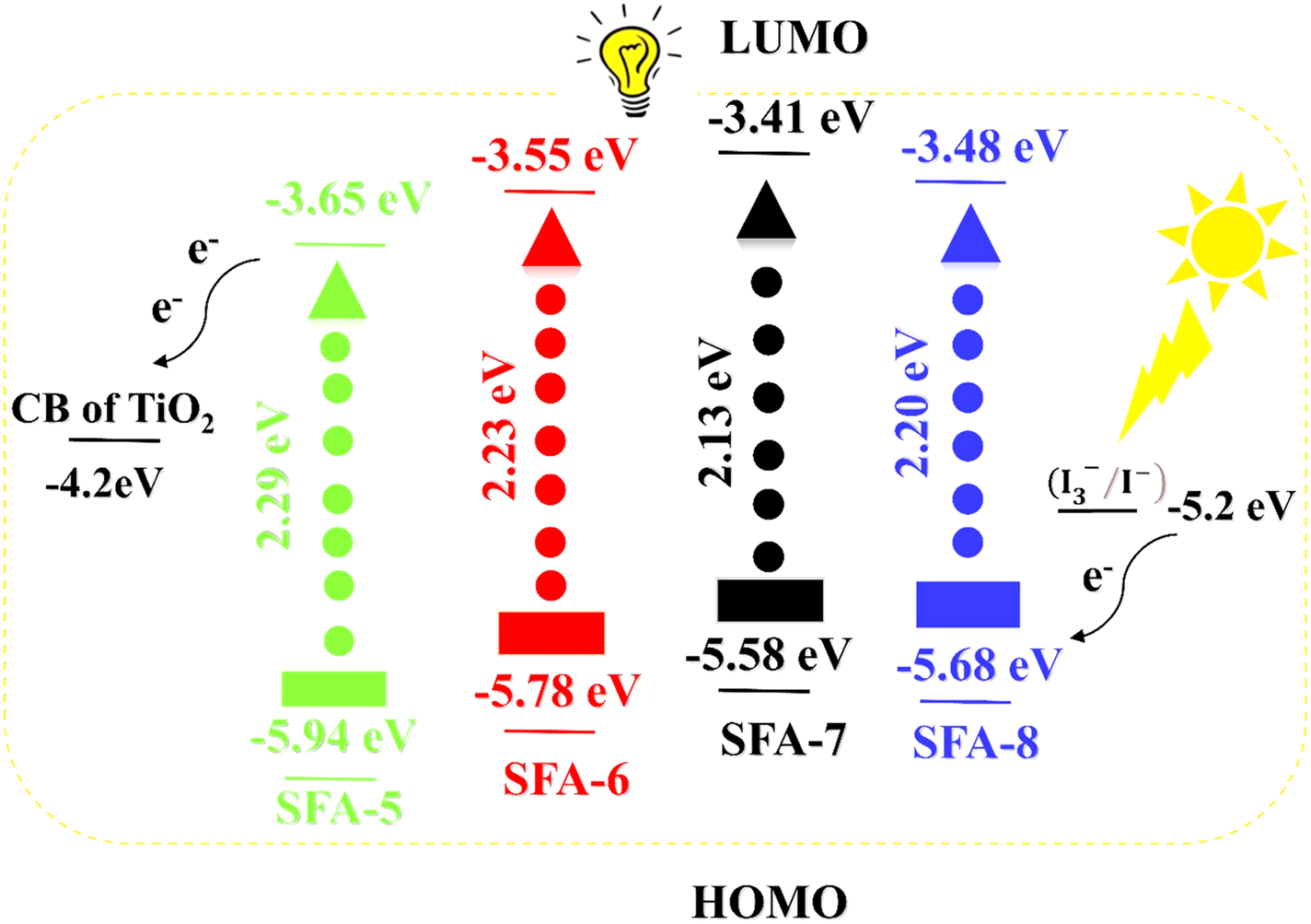
Energy level diagram for **SFA-5–8** sensitizers.

Cyclic voltammetry (CV) experiments were conducted to determine the electron transfer feasibility from the **SFA-5–8** sensitizers to the TiO_2_ molecule, as well as to investigate the electron regeneration of the dyes via calculating the ground and excited oxidation potentials of the newly synthesized sensitizers^42^ **(SFA-5–8**). These values represent the key criterion to evaluate the suitability of an organic dye for DSSC applications and provide a deeper insight into the thermodynamic driving force of electron injection and dye regeneration. Such corresponding values are depicted in Fig. 7, GSOP and ESOP were calculated as stated in Eq. (10), where the oxidation onset stands for the onset oxidation potential of CV oxidation peak^42^. GSOP values for the studied **SFA-5–8** dyes were found to be: **SFA-5 **(− 5.94 eV), **SFA-6** (− 5.78 eV), **SFA-7** (− 5.58 eV), and **SFA-8 (**− 5.68 eV). The HOMO of sensitizers is more negative than the value of ${(\text{I}}_{3}^{ -}/{\text{I}}^{-})$ (− 5.2 eV) redox electrolyte energy level, respectively, thus confirming the possibility of the dye regeneration^43^.

10 $$ {\text{ESOP}} = \left[ {\left( {{\text{GSOP}}\left( {{\text{eV}}} \right) + {4}.{7}} \right) - E_{{0{-}0}} } \right]{\text{ eV}} $$

Additionally, ESOP of sensitizers **(SFA-5–8)** was estimated from the above-mentioned equation using both the GSOP and *E*_*0-0*_ (calculated from the absorption spectra of the sensitizer). The ESOP energy levels are lying between − 3.48 and − 3.65 eV for the sensitizers: **SFA-5** (− 3.65 eV), **SFA-6** (− 3.55 eV), **SFA-7** (− 3.41 eV), and **SFA-8** (− 3.48 eV), respectively. Those values are higher than that of the TiO_2_ conduction band. It is worth mentioning that the LUMO level of the di-anchoring dyes completely changed for the mono-anchoring counterparts, associated with the conjugation length and the electron-withdrawing character of the synthesized dyes acceptors as well as the HOMO–LUMO minimized band gaps^44^. As expected **SFA-6–8** molecules with nitrocyanoacetamide, carboxycyanoacetamide, and pyridinyl cyanoacetamide as strong acceptor moieties showed the smallest bandgap values compared to that of **SFA-5** with the phenylacetonitrile segment. That could be a direct evidence on the highest degree of conjugation between the electron-accepting (CO, CN, NO_2_, COOH, pyridyl ring) units and donor parts. As could be noticed, all ESOP data are energetically less positive than that of the TiO_2_ CB potential (− 4.2 eV), unraveling the efficient injection of the electron to the TiO_2_ edge. Amongst all synthesized dyes containing cyanoacetamide segments, **SFA-7** contains the strongest electron-withdrawing group (COOH) than pyridyl cyanoacetamide of **SFA-8** and 4-nitrocyanoacetamide in **SFA-6**. That in turn could imply a deficient driving force for the electron transfer by possessing the smallest *E*_*0-0*_ values and ensuring efficient light capture. All the sensitizers **(SFA-5–8)** could meet the thermodynamics prerequisite, hence rendering them suitable to be used as effective sensitizers for TiO_2_-based DSSCs.

#### Molecular modeling

**Figure 8:**
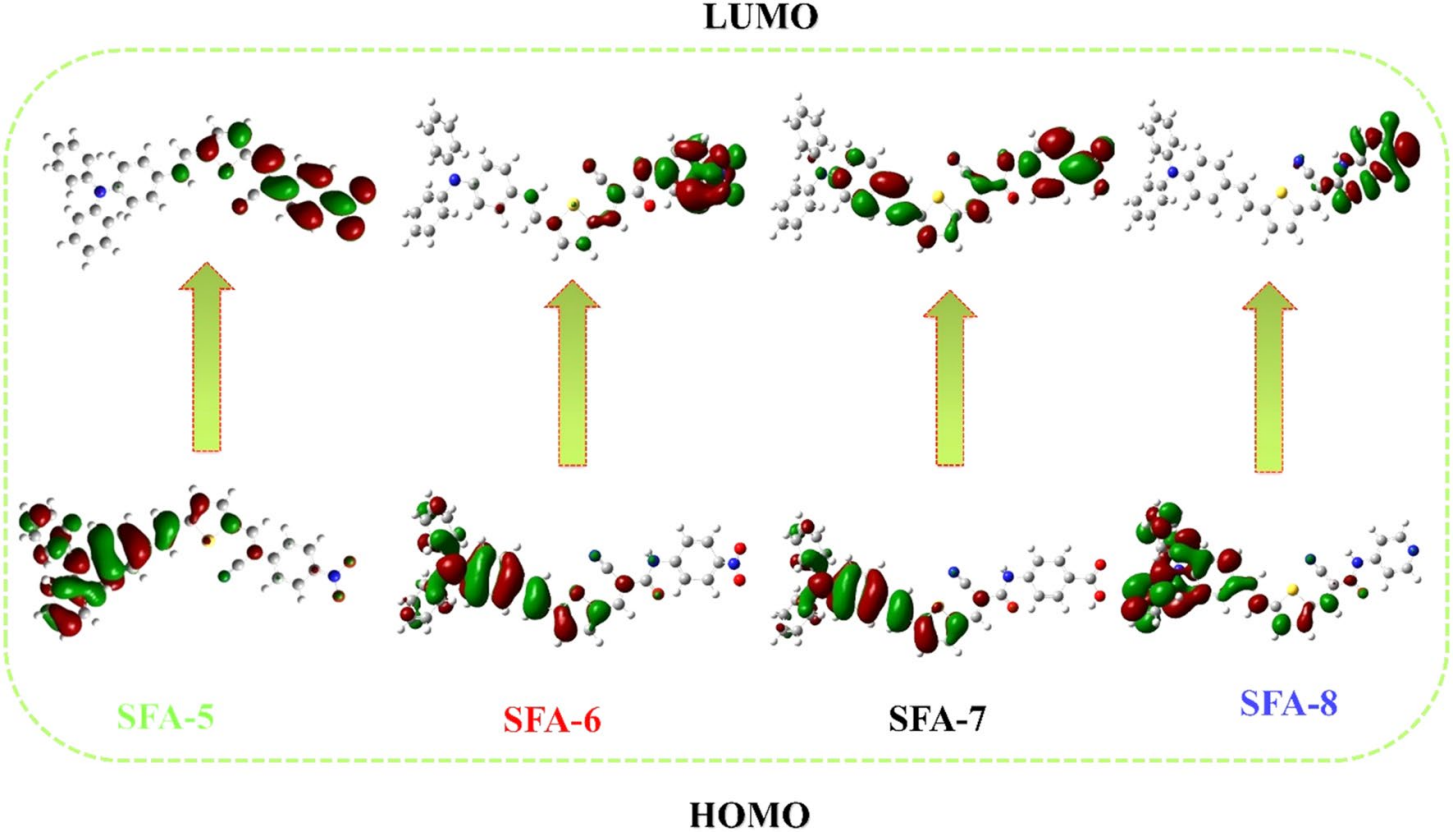
Optimized geometry structures for **SFA-5–8** sensitizers.

DFT calculations were performed on **SFA-5–8** sensitizers using the B3LYP hybrid method in parallel with the d-polarized 6–311G basis implemented in the Gaussian09 program^37^. Figure 8 gives more insights into the relationship between geometric structure and electronic distribution of the HOMOs and LUMOs levels for the four sensitizer dyes^45^. Intramolecular charge transfer (ICT) from the dye HOMO level (depending on donor part) to its LUMO level (depending on acceptor part) is considered one of the most important features for achieving effective charge separation and better electron injection. For **SFA-5–8** sensitizers, there is an effective charge separation between the HOMOs and the LUMOs levels. For the **SFA-5** sensitizer, the electron density of HOMO was primarily localized on the donor parts (triphenylamine and thiophene ring), whereas the electron density of LUMO was largely localized on 4-nitroacetonitrile acceptor moiety (CN and NO_2_). However, this case was different for the **SFA-6** as the 4-nitrocyanoacetamide insertion had enlarged the conjugative system without impairing the coplanarity of the entire molecule. As such, the HOMO electron density was localized on triphenylamine donor part, but the LUMO counterpart shows a clear shift in the electron density towards the acceptor moieties (CN, CO, and NO_2_). Noteworthy, the introduction 4-carboxylcyanoacetamide into the **SFA-7** dye could facilitate the electron transfer from the TPA donor side and thiophene moieties to the acceptors part that localized on CN, CO, and COOH segments. As for the **SFA-8**, the electronic distribution of its HOMO is mainly distributed over the donor and π-spacer, however, its LUMO orbitals are distributed mainly on the electron acceptor units of the 4-pyridylcyanoacetamide, resulting in strong electronic interaction with TiO_2_ surface. It is not surprising to mention that the intramolecular charge transfer (ICT) from various donors to phenyl acrylonitrile/2-cyanoacetamide donors across all sensitizer moieties via the π-spacer had facilitated the HOMOs-LUMOs spatial level separation upon light irradiation. Accordingly, the electron injection from the excited sensitizer dyes to the TiO_2_ surface had significantly enhanced.

#### Molecular electrostatic potential (MEP)

**Figure 9:**
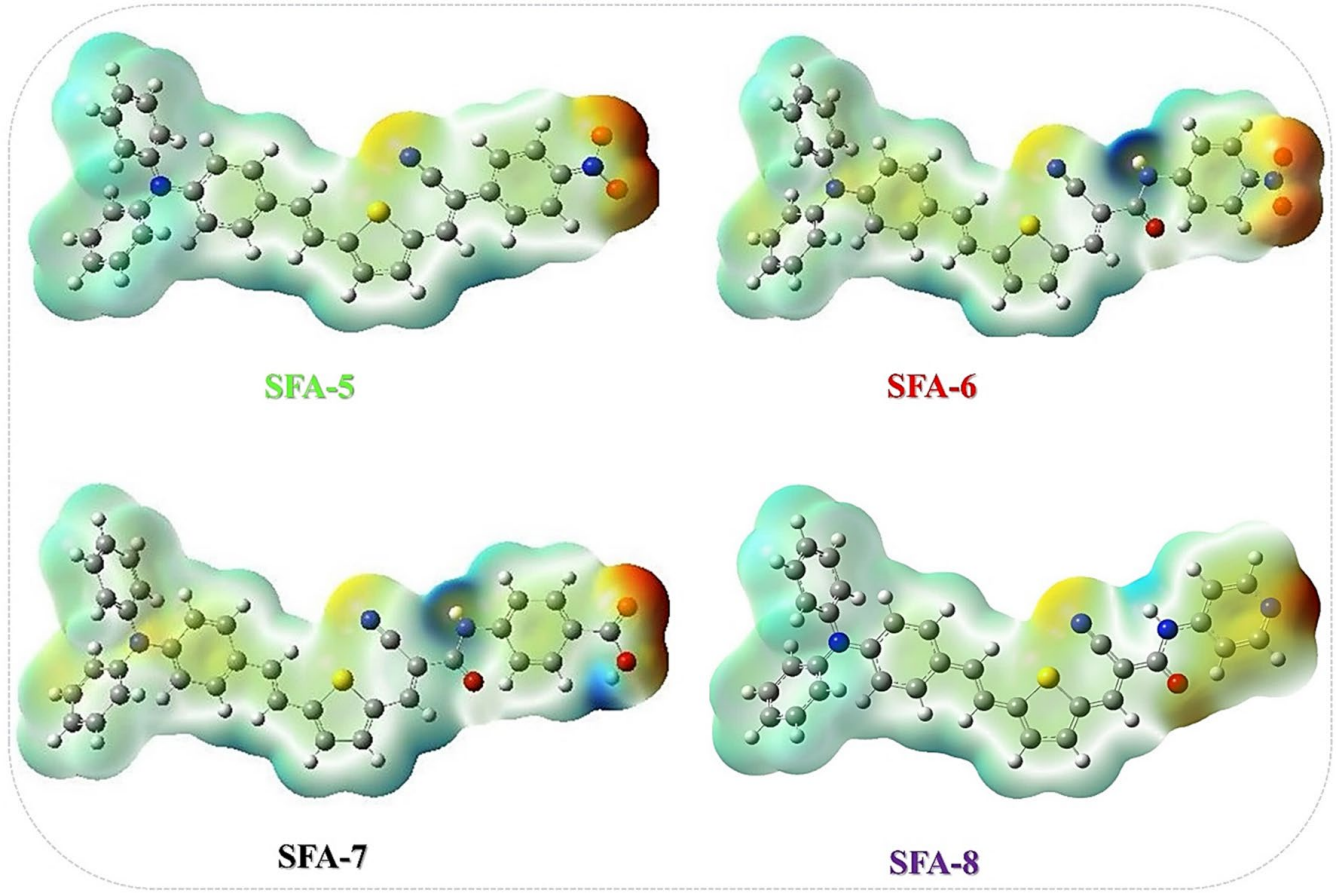
Molecular electronic potential diagram (MEP) of **SFA-5–8** sensitizers.

Molecular electrostatic potentials (MEP) is one of the efficient approaches to identify the internal charge transfer (ICT) property of the entire organic molecules between HOMO–LUMO levels^46^ of triphenylamine of **SFA-5–8** dyes, which can be obtained from the cube file of the Gaussian job^37^. The effect of donor–acceptor groups was analyzed by inspecting the different HOMO–LUMO levels and (MEPS) of all **SFA-5–8** sensitizers (Fig. 9). On one hand, the negative (red) low potentials of **SFA-5** are found prominently around the region of the anchoring group which is concentrated on carbonyl, cyano (CN), and (NO_2_) groups. On the other hand, the negative charge of **SFA-6** molecules with cyanoacetamide moieties were localized over cyano, carbonyl, and nitro groups. As for the **SFA-7** dye, its negative charge was localized on the cyano group, and carbonyl attached to the COOH group. Furthermore, the negative charge of **SFA-8** is founded to be linked to CN, CO, as well as the nitrogen of the pyridine ring. From further insights, the blue positive region of the MEP map was localized over the donor moieties such as the triphenylamine ring, π- conjugation system, and thiophene ring regions, thus demonstrating their favorite sites for nucleophilic attack. Indeed, analyzing all previous MEP features of all synthesized **SFA-5–8** sensitizers revealed the electron feasibility for possible interaction with another group of atoms. These findings can reasonably give an indication of the ICT nature of all synthesized **SFA-5–8** sensitizers when they are adsorbed on the TiO_2_ surface.

#### *TiO*_*2*_* electrode preparation and device fabrication*

TiO_2_ fabrication process was thoroughly provided in the “Supplementary information file (SI)”.

#### Photovoltaic device characterizations

**Figure 10:**
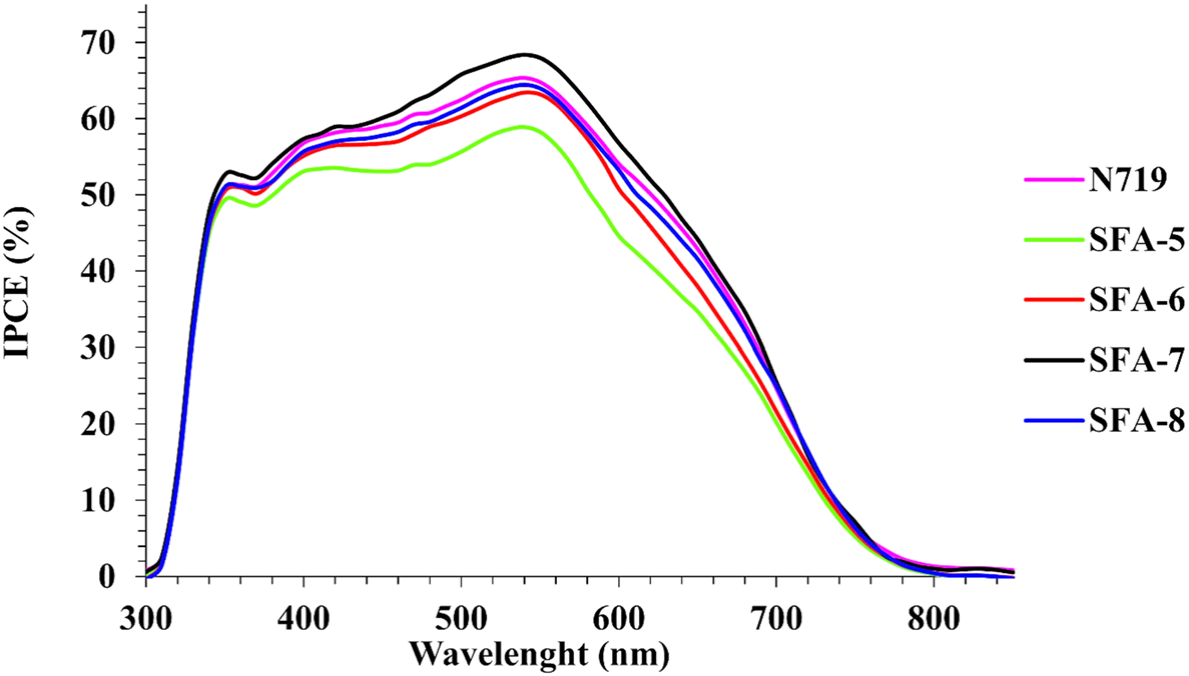
Incident-photon-to-current conversion efficiency *(IPCE)* spectra for all studied **SFA-5–8** sensitizers.

To further evaluate the Photovoltaic enhancement and assess the electron injection efficiency of all sensitized devices **(SFA-5–8)** side by side with the charge carrier efficiency of the adsorbed dyes on the TiO_2_ surface, the incident photon-to-electron conversion efficiency *(IPCE)* was extensively measured under no external bias, as depicted in Fig. 10. That in turn could be a very vital tool for establishing the structure–property relationship, determining the best electron anchoring group for TPA-sensitizers systems, and more importantly, linking the adsorber structure with their corresponding solar performance^47^. As shown in Fig. 10, *IPCE* values of all DSSC based on **SFA-5–8** sensitizers showed maximum *IPCE* peaks reaching (58–68%) at a range of 300–800 nm, confirming the vital role of pheylacetonitrile and 2-cyanoacetamide derivatives present in all synthesized dyes on enhancing the photoelectrochemical performance. Specifically, **SFA-7** reported the highest *IPCE* peak of above 68% at ~ 300–800 nm along with the enhanced *J*_*SC*_ value amongst all studied dyes. This can be explained based on the fact that incorporating a strong electron-withdrawing carboxylic group (COOH) of the 4-carboxylcyanoacetamide segment to other anchoring CO and CN moieties of **SFA-7** contributed largely to maintaining the highest *IPCE* value. That was even higher than those reported for **N719 dye**, thus revealing higher photocatalytic efficiency of 4%. As for the **SFA-8** sensitizer, carboxylic group replacement with a pyridyl ring has minimized its electron light-harvesting ability and hindered its electron injection, thus reporting a lower efficiency in contrast to the **SFA-7** sensitizer. Although 4-nitrocyanoacetamide **SFA-6** has the same skeleton of carboxyl and pyridyl 2-cyanoacetamide sensitizers as **SFA-7–8** analog, the *IPCE* peak of the **SFA-6** was found to be lower than its corresponding **SFA-7** and **SFA-8** sensitizers, mainly highlighting the strength of the electron-withdrawing groups^48^. This lower efficiency could be attributed to the presence of nitro group (NO_2_) that strongly promoted the dye aggregation and hindered the *IPCE* values, as confirmed in Fig. 10. Predictably, cells sensitized by **SFA-5** offered weaker absorption with a less covered surface on the TiO_2_ surface compared to other **SFA-6–8** sensitizers, thus influencing the overall yield and retard the electron injection processes^49^. The *J*_*sc*_^*IPCE*^ values integrated from the *IPCE* spectra are in good consistency with the *Jsc* values measured in the *J*–*V* measurements. Consequently, the cell with dye **SFA-7** gives the highest and broadest *IPCE* spectrum, confirming its highest *Jsc.* The improved IPCE responses are consistent with the improved *Jsc*^49^. Furthermore, the *IPCE* spectra of all **SFA-5–8** sensitizers and **N719** are in good agreement with their UV–vis absorption spectra on the TiO_2_ surface.

**Figure 11:**
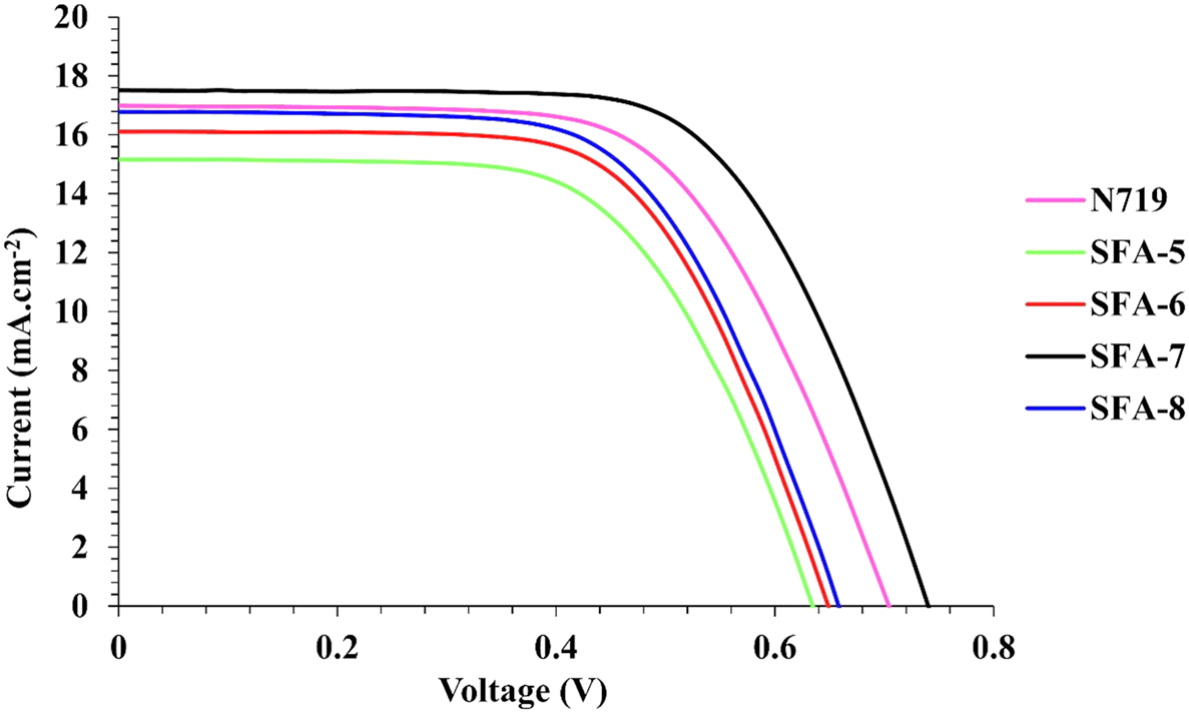
Current density–voltage characteristics for DSSCs based **SFA-5–8** and **N-719**.

Table 4 summarizes all corresponding photovoltaic parameters including; short-circuit photocurrent density (*J*_*SC*_), open-circuit photovoltage (*V*_*OC*_), fill factor (*FF*), and power conversion efficiency *(ηcell).* The photocurrent density–voltage spectra for **N719**, phenylacetonitrile (**SFA-5**), and 2-cyanoacetamide sensitizers (**SFA-6–8**) are depicted in Fig. 11. As could be inferred, the enhanced *IPCE* response of 4-carboxylcyanoacetamide **SFA-7** sensitizer was translated into the highest *Jsc* of approximately (17.51 mA/cm^2^) in comparison with the reported *Jsc* values of **N719** (17.00 mA/cm^2^), **SFA-8** (16.78 mA/cm^2^), **SFA-6** (16.10 mA/cm^2^), and **SFA-5** (15.16 mA/cm^2^) counterparts. Such short circuit current enhancement of **SFA-7** molecule could be mainly linked to better electron injection efficiency to TiO_2_ conduction band compared to other sensitizers. That was also confirmed by the highest value of its LUMO levels. In a bid to further examine the photovoltaic performance of the synthesized cells, open-circuit voltage and fill factor could give insights into the photoelectrochemical performance of the synthesized sensitizers. For instance, **N719** and **SFA-5–8** dyes yielded power conversion efficiency (% *ηcell*) of 7.25 (*Voc* = 0.70 V and *FF* = 0.61), 5.53 (*Voc* = 0.63 V and *FF* = 0.69), 5.77 (*Voc* = 0.64 V and *FF* = 0.56), 7.56 (*Voc* = 0.72 V and *FF* = 0.60), and 6.43 (*Voc* = 0.65 V and *FF* = 0.59), respectively. The *V*_*OC*_ of sensitizers follows the order of **SFA-7** > **N719** > **SFA-8** > **SFA-6 > SFA-5**.

**Table 4:** Photovoltaic parameters of all studied **SFA-5–8** sensitizers; data are pooled for three devices and summarized as mean ± standard deviation (The best device parameters, which listed in the manuscript).

Sensitizer (0.2 mM)	*V*_*OC*_ (V)	*J*_*SC*_ (mA cm^−2^)	*J*_*SC*_^*IPCE*^ (mA cm^−2^)	*FF*	*ηcell* (%)
N719	0.67 ± 0.03 (0.70)	16.96 ± 0.04 (17.00)	16.95	0.59 ± 0.01 (0.61)	7.21 ± 0.02 (7.25)
SFA-5	0.62 ± 0.01 (0.63)	15.14 ± 0.02 (15.16)	13.99	0.67 ± 0.02 (0.69)	5.51 ± 0.03 (5.53)
SFA-6	0.62 ± 0.03 (0.64)	16.05 ± 0.06 (16.10)	15.86	0.54 ± 0.01 (0.56)	5.69 ± 0.13 (5.77)
SFA-7	0.71 ± 0.02 (0.72)	17.49 ± 0.01 (17.51)	16.95	0.58 ± 0.03 (0.60)	7.32 ± 0.25 (7.56)
SFA-8	0.63 ± 0.03 (0.65)	16.77 ± 0.02 (16.78)	16.27	0.56 ± 0.04 (0.59)	6.36 ± 0.13 (6.43)

As stated in Table 4, **SFA-5**, the anchoring occurs through the cyano group (CN) working as a withdrawing group, and the coordination between the nitro group and the TiO_2_ surface causes optical bleaching of the light absorption, increases the dye/TiO_2_ coupling, which provides the process of anchoring on the surface TiO_2_. Nitrophenyl acetonitrile sensitizers (**SFA-5**) showed the lowest efficiency attributed to the weakest acceptor moieties compared to other 2-cyanoacetamides sensitizers (**SFA-6–8)** and showed the lowest values of *J*_*SC*_ and *V*_*OC*_ values. For **SFA-6**, by replacing the phenyl acetonitrile by 4-nitrocyanoacetamide, the anchoring process occurs through a carbonyl group (CO), (NH), (NO_2_), and (CN) groups that work as excellent withdrawing groups which provide the process of anchoring on the surface TiO_2_, increasing the number of anchoring function groups enhanced the efficiency than anchoring by only (CN) and (NO_2_) in **SFA-5**, the highest *J*_*SC*_, and better *V*_*OC*_ values were reported for the 4-carboxylcyanoacetamide **SFA-7** sensitizer associated with a higher photoconversion efficiency of about 7.56%, thus outperforming the best-reported dyes which mainly related to the various strong acceptor's group, carboxyl group plays a great role in surface adsorption on TiO_2_ addition to act as electron acceptors, given excellent electron-withdrawing capabilities relative to the Brønsted acid sites employed for the adsorption of carboxylates_._ The presence of a second strong anchoring group (COOH) as an extra acceptor addition to the various anchors across the 2-cyanoacetamide segment (CO), (CN), and (NH) act as bifunctionality of acceptor and electron-withdrawing groups which seems to enhance the performance of the sensitized cells and the electron injection into the conduction band of TiO_2_ through *H*-aggregates^50^. This is not surprising as the photoconversion efficiency of the **SFA-7** sensitizer was raised by 4% with respect to **N719**. Finally, for **SFA-8**, using 4-pyridylcyanoacetamide for the first time as a new bifunctionality anchoring and electron withdrawing system, by the formation of coordination bonds between the nitrogen atom in the pyridyl ring of the Lewis acidic sites of the TiO_2_ surface leads to efficient electron injection, addition to the electron-withdrawing groups across the 2-cyanocetamide segment (CO, NH and CN groups). Moreover, **SFA-6** and **SFA-8** sensitizers carrying 2-cyanoacetamide segments showed lower efficiency than **SFA-7**, which mainly related to the strength of the anchoring group in the order of COOH > pyridyl > NO_2_. Thus, *Jsc* and *V*_*OC*_ values have been changed accordingly. Based on the above findings, it could be noticed that the experimental results are in accordance with the theoretical predictions.

#### Electrochemical impedance spectroscopy (EIS)

**Figure 12:**
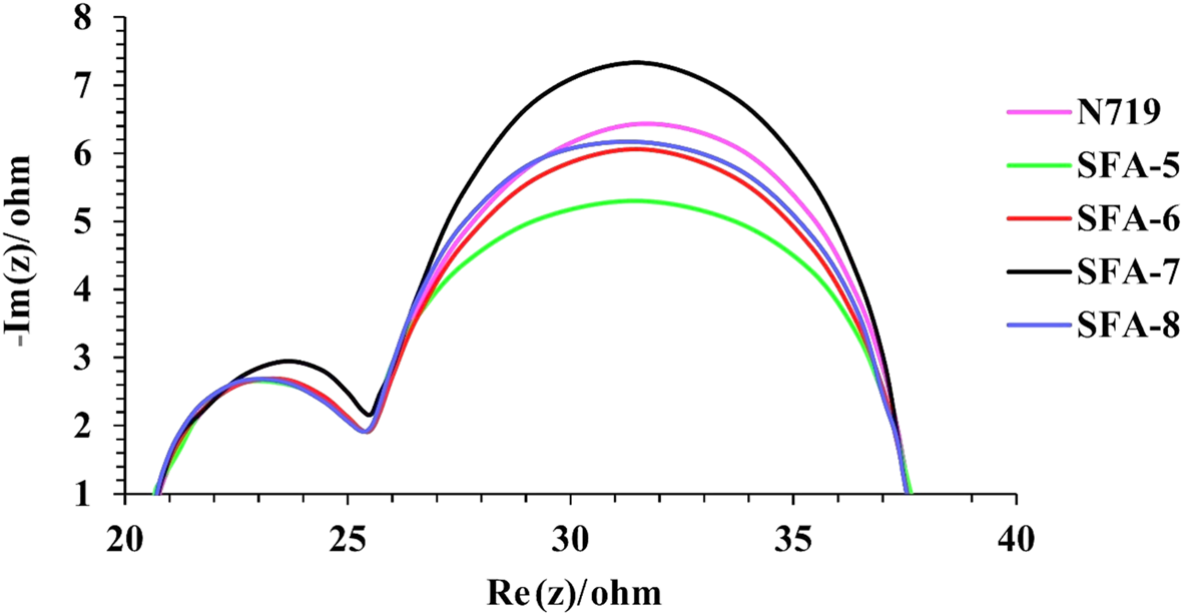
EIS Nyquist plots for DSSC sensitized with **SFA-5–8**.

To better correlate the charge dynamic characteristics of all synthesized **SFA-5–8** sensitizers with the enhanced photocatalytic performance, electrochemical impedance spectroscopy (EIS) was investigated to effectively determine the charge transfer resistance (R_CT_) and the interfacial capacitance at the TiO_2_ electrode/dye/electrolyte and Pt/electrolyte interfaces^51^. Figure 12 shows the Nyquist plots of all sensitized cells based on phenylacetonitrile, 2-cyanoacetamide **SFA-5–8 and N719** sensitizers. As depicted in Fig. 12, EIS data showed two distinct semicircles that were properly fitted using simplified Randle’s equivalent circuit. A small semicircle observed in the low-frequency region could represent the cathode charge transfer resistance which is directly related to *FF*. On the other hand, a large semicircle was observed in the middle frequency regime that could be originated from charge transfer resistance *(R*_*CT*_*)* from TiO_2_ molecule to the electrolyte solution, which is directly related to the *V*_*oc*_. According to Nyquist plots, the semicircle radius of all sensitized **SFA-5–8** cells follow the order of **SFA-7 > N719 > SFA-8 > SFA-6 > SFA-5.** It is worth noting that the *R*_rec_ of 4-carboxylcyanoacetamie **SFA-7** is larger than that of the **N719** counterpart, indicating that the charge carriers recombination process was retarded upon sensitization. In addition, the high *R*_rec_ value of the **SFA-7** molecule upon sensitization could be mainly ascribed to the **SFA-7** structure containing various acceptors and anchoring moieties that are favorable for retarding charge recombination process. To this end, Nyquist plots unravel that sensitization by 2-cyanoacetamide sensitizers could be beneficial for reducing dark current and suppressing charge recombination at the TiO_2_/dye/electrolyte interface^52^.

**Figure 13:**
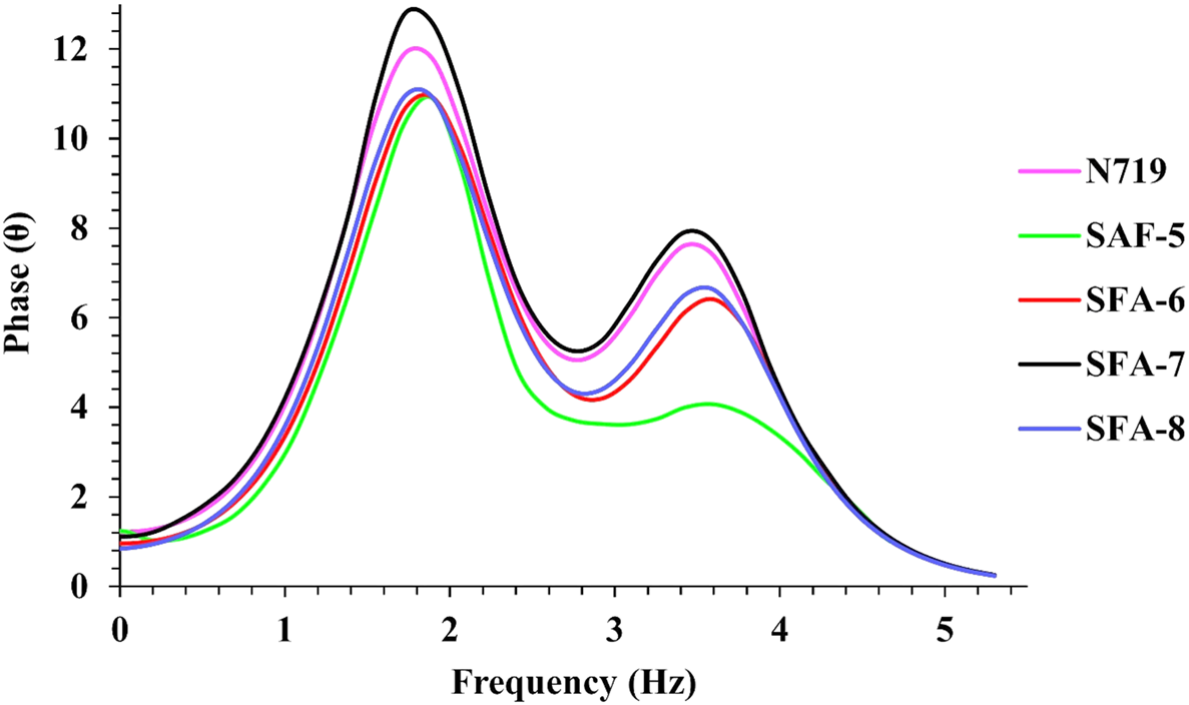
EIS Bode plots for DSSC sensitized with **SFA-5–8**.

On the other hand, EIS could be represented using Bode frequency plots, as illustrated in Fig. 13. That was estimated using the following equation; (τ_eff_ = 1/2π*f*)^52^, where t represents the electron lifetime injected into TiO_2_ and *f* is the mid-frequency peak in bode plots, which is directly related to the electron lifetimes. Notably, the electron lifetime that is mainly related to *Voc* was determined for all synthesized **SFA-5–8** molecules using Bode frequency plots. The values of the mid-frequency peaks of the bode plots followed the order of **SFA-7 > N719 > SFA-8 > SFA-6 > SFA-5,** corresponding to electron lifetimes in the sequence of **SFA-5** < **SFA-6** < **SFA-8 < SFA-7**. Similarly, the corresponding electron lifetimes were found to be 4.48, 3.25, 2.58, 2.14, and 1.26 ms, respectively, in agreement with *V*oc values. For the sensitized **SFA-5–8** and **N719** dyes, the electron lifetimes follow the order of **SFA-7 > N719 > SFA-8 > SFA-6 > SFA-5**, respectively, which lead to a significant enhancement in photocurrent and photovoltage and considerably higher cell efficiency^53^.

## Conclusion

In summary, we have synthesized four innovative phenylacetonitrile and 2-cyanoacetamide derivatives of dyes **SFA-5–8** with different auxiliary donors and acceptors. We studied how the presence of a thiophene spacer and different auxiliary acceptors (NO, CO, CN, COOH, and pyridyl groups) affected the photophysical and electrochemical performance of DSSC devices. DFT and TD-DFT were used to explore the photophysical and electronic structures of all studied **SFA-5–8** organic dyes. The molecular orbital energy levels of **SFA-5** sensitizers possessed an appropriate thermodynamic driving force that was sufficiently suitable for electron injection from the LUMOs into the TiO_2_ conduction band. The calculated studies of the dihedral angle and NBO of **SFA-5–8** revealed an efficient ICT across sensitizers from the donor to acceptor parts. The higher electron acceptance and lower chemical hardness values of **SFA-5–8** molecules suggested excellent photoelectric conversion performance. Further, we measured the photovoltaic properties to properly investigate the direct effect of various anchoring units on the overall performance of the DSSCs. The overall photoconversion efficiency of DSSCs for all **SFA-5–8** sensitizers was approximately 5.53–7.56%. The number of sensitizers loaded into the TiO_2_ surface is in accord with the *PCE* values. Remarkably, **SFA-7** showed superior photoconversion efficiency associated with the highest *Jsc* and *Voc* which could be attributed to reduced recombination rates of charge carriers and the increased electron lifetime. The sensitizations of the new **SFA-5–8** sensitizers were investigated in comparison to the **N719** dye. Interestingly, the optimized **SFA-7** attained a higher *PCE* by approximately 4% over that of **N719** sensitized one. This splendid activity can be ascribed to the excess anchoring groups of the synthesized dyes that could lead to a longer blocking layer that minimizes charge recombination. Such breakup of π-stacked aggregates could improve the electron injection yield, rendering **SFA-5–8** sensitizers superior candidates for applied DSSC applications.

## Supplementary Information

Supplementary Information.

## Data Availability

The datasets used and/or analyzed during the current study available from the corresponding author on reasonable request.
