# Supplementary material for: Synthesis of innovative triphenylamine-functionalized organic photosensitizers outperformed the benchmark dye N719 for high-efficiency dye-sensitized solar cells
Source: Sci Rep. 2022 Jul 28;12:12885. doi: 10.1038/s41598-022-17041-1 (PMC9334599; doi:10.1038/s41598-022-17041-1)
Supplement: Supplementary file 1 — Supplementary Information. [file 41598_2022_17041_MOESM1_ESM.docx]

**Supplementary file**

**Synthesis of Innovative Triphenylamine-Functionalized Organic Photosensitizers Outperformed the Benchmark Dye N719 for High-Efficiency Dye-Sensitized Solar Cells**

Safa A. Badawy, Ehab Abdel-Latif, Ahmed A. Fadda, Mohamed R. Elmorsy^🖂^

*Department of Chemistry, Faculty of Science, Mansoura University, 35516 Mansoura, Egypt, ^🖂^E-mail: m.r.elmorsy@gmail.com*

**1. Analytical Measurements**

**Figure S1. UV-Vis. absorption of N719**

| **λ_max_ (nm)** | **ε (10^4^M^-1^ cm^-1^)** | **λ_onset_ / nm** | ***E*_0-0_ (ev)** |
| --- | --- | --- | --- |
| **307, 384, 537** | **3.69, 1.18, 1.08** | **602** | **1.92** |


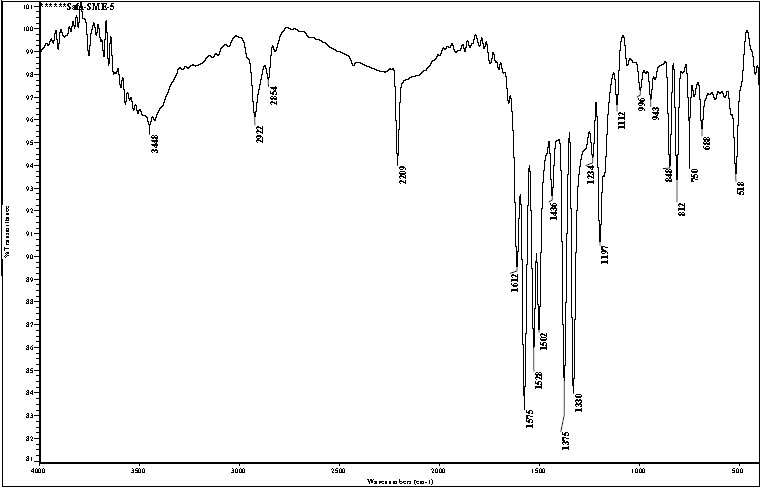

**Figure S2. IR spectrum of senstizer SFA-5.**


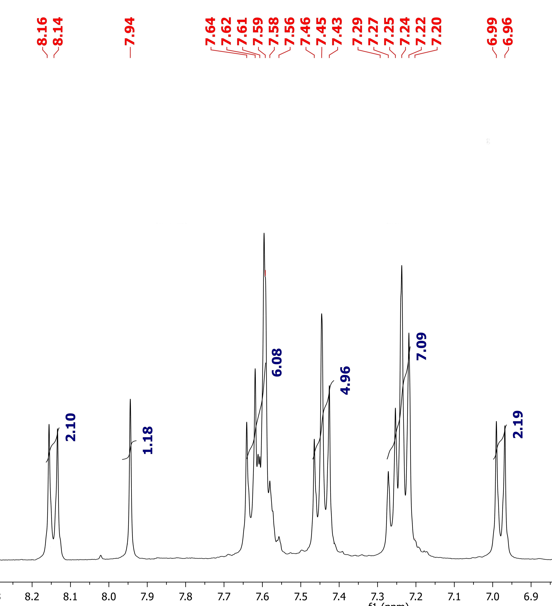

**Figure S3. ^1^H NMR spectrum of sensitizer SFA-5.**


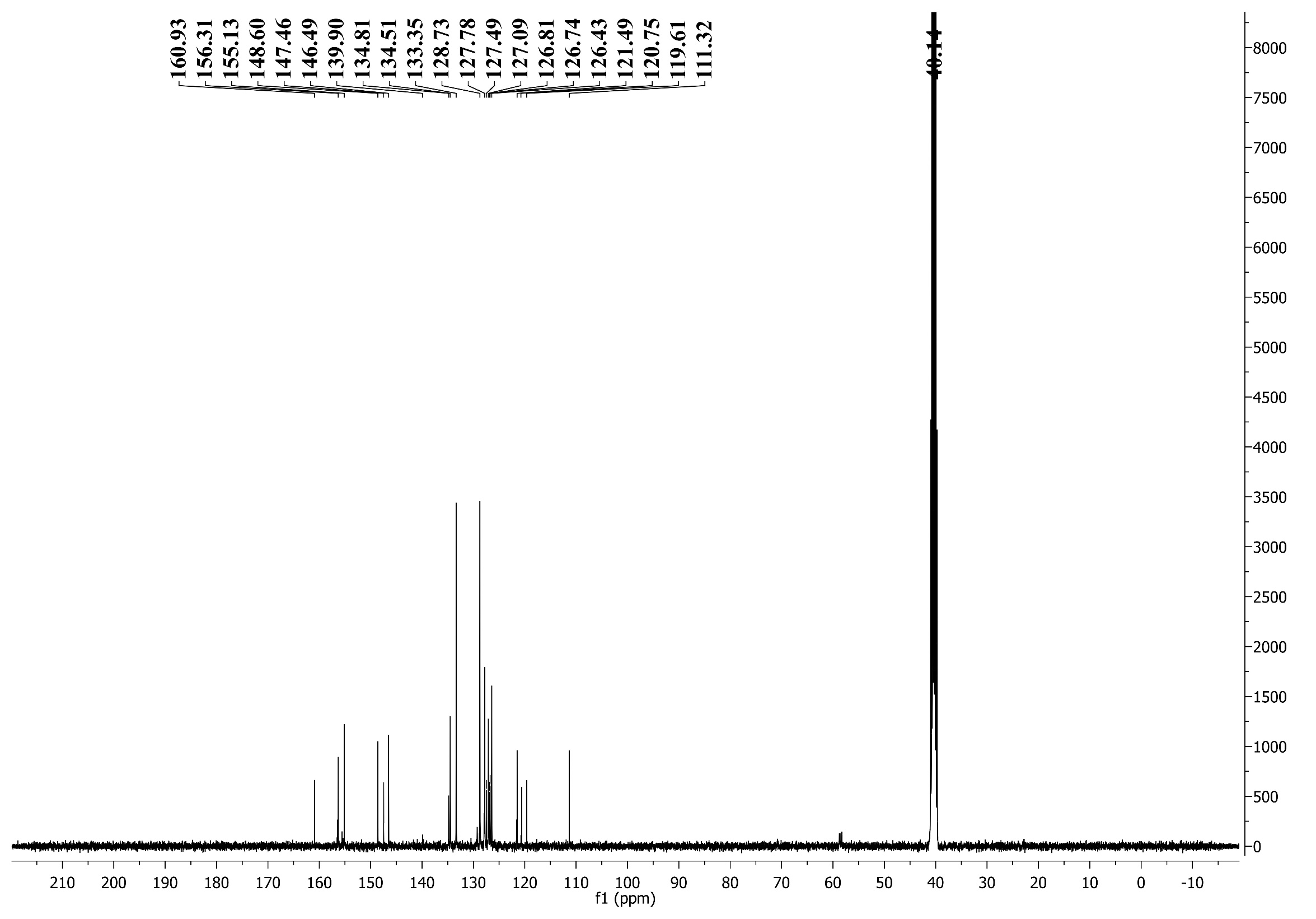

**Figure S4. ^13^C NMR spectrum of sensitizer SFA-5.**


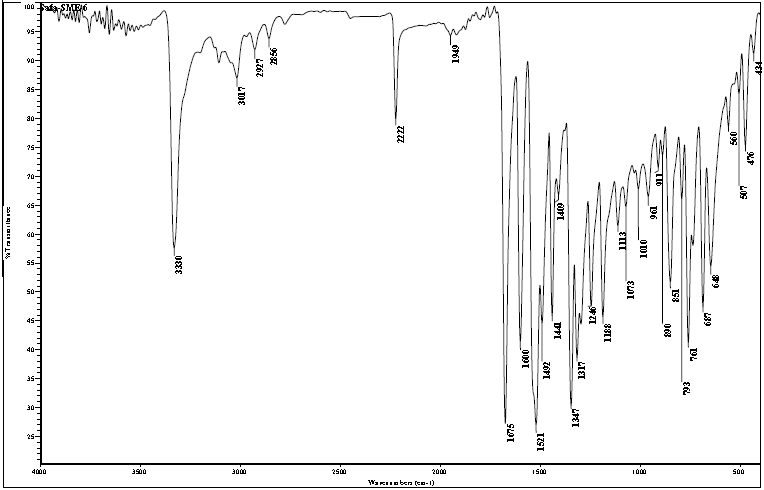

**Figure S5. IR spectrum of senstizer SFA-6.**


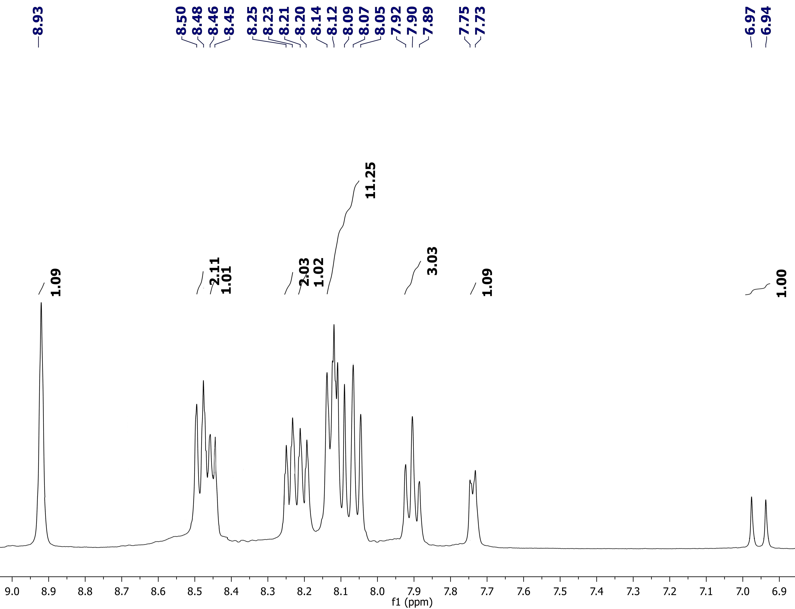

**Figure S6. ^1^H NMR spectrum of sensitizer SFA-6.**

**
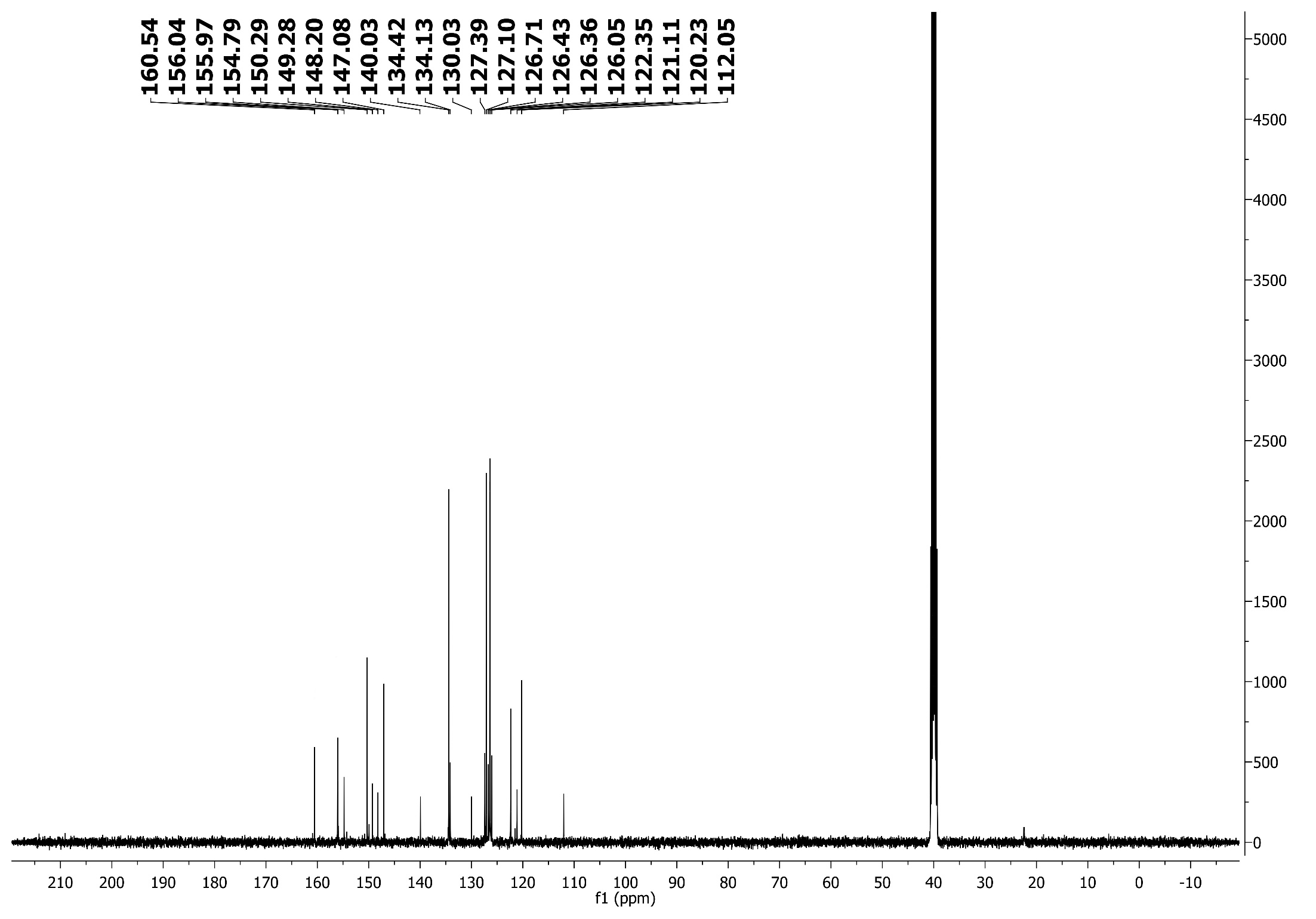
**

**Figure S7. ^13^C NMR spectrum of sensitizer SFA-6.**


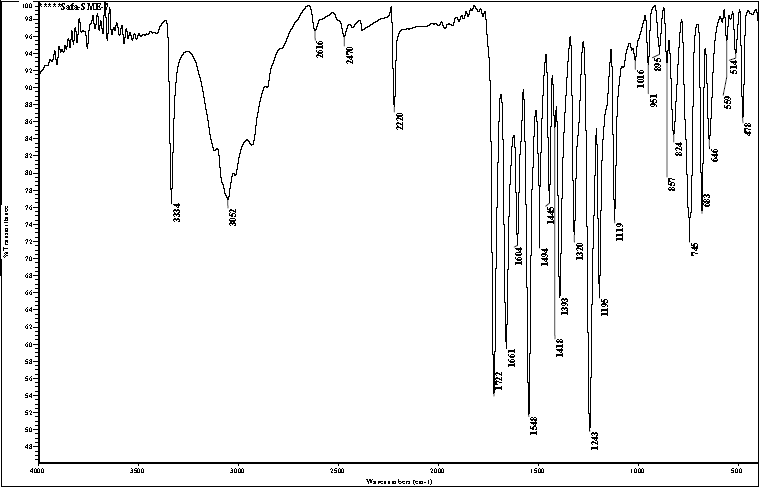

**Figure S8. IR spectrum of senstizer SFA-7.**


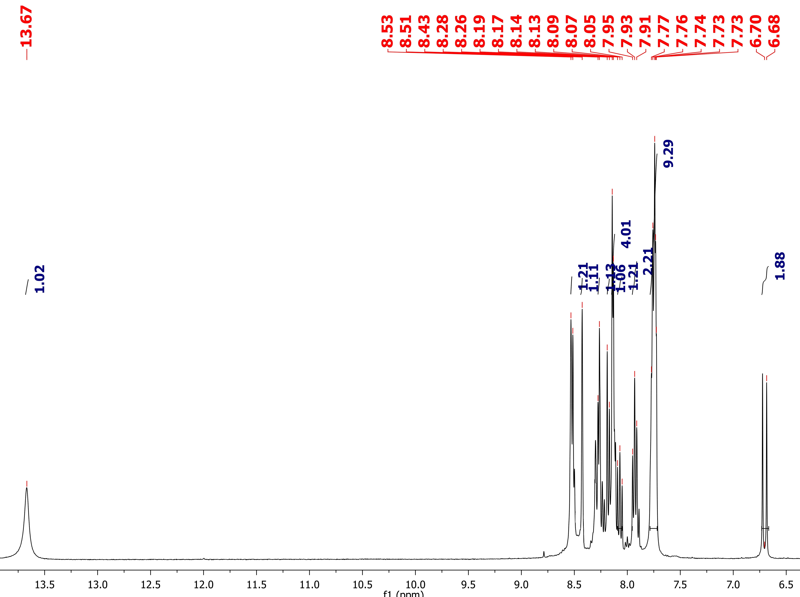

**Figure S9. ^1^H NMR spectrum of sensitizer SFA-7.**

**
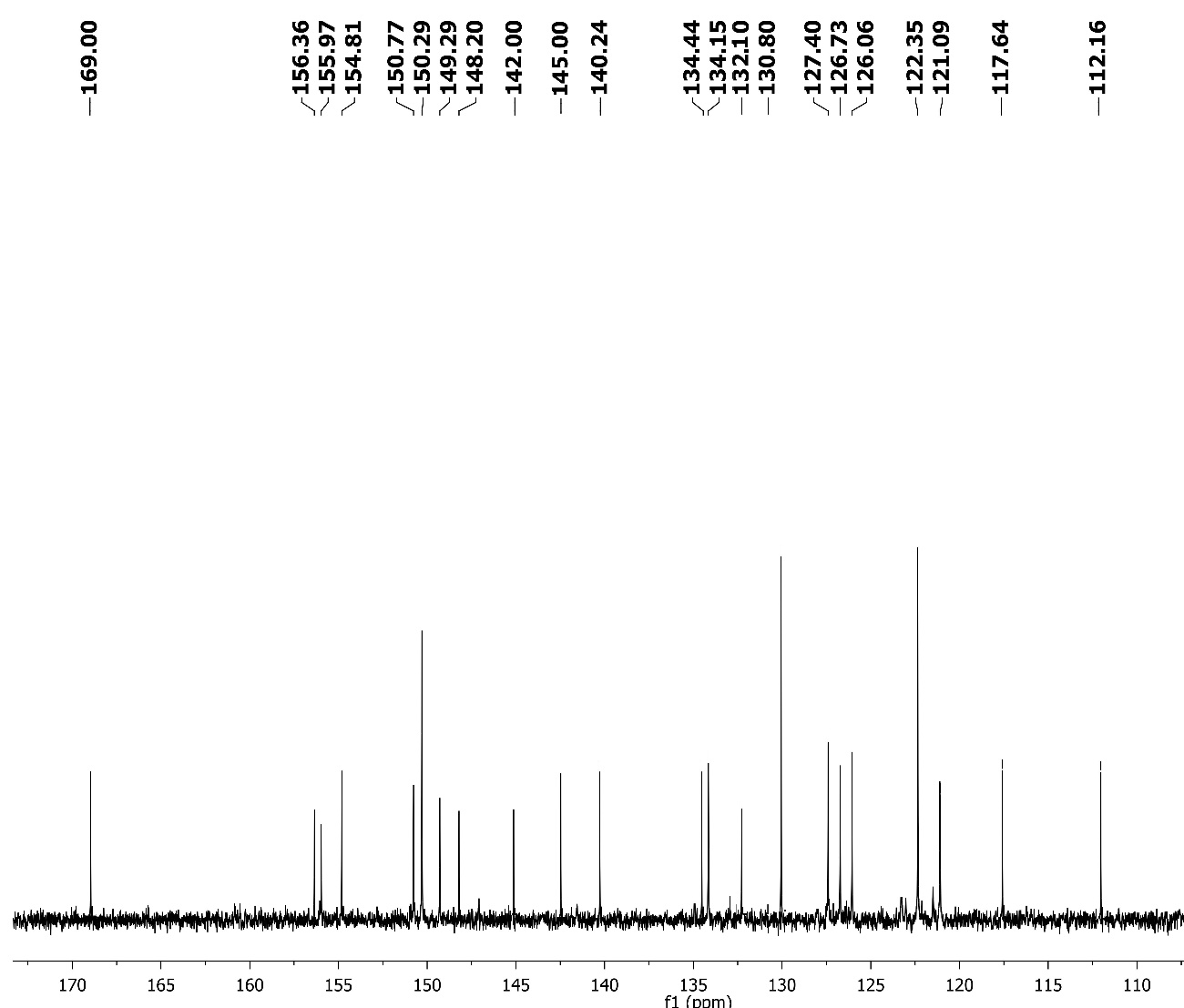
**

**Figure S10. ^13^C NMR spectrum of sensitizer SFA-7.**


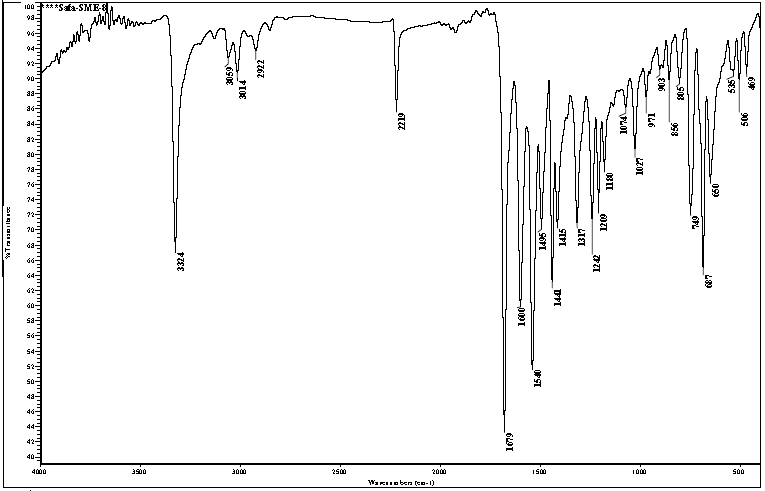

**Figure S11. IR spectrum of senstizer SFA-8.**


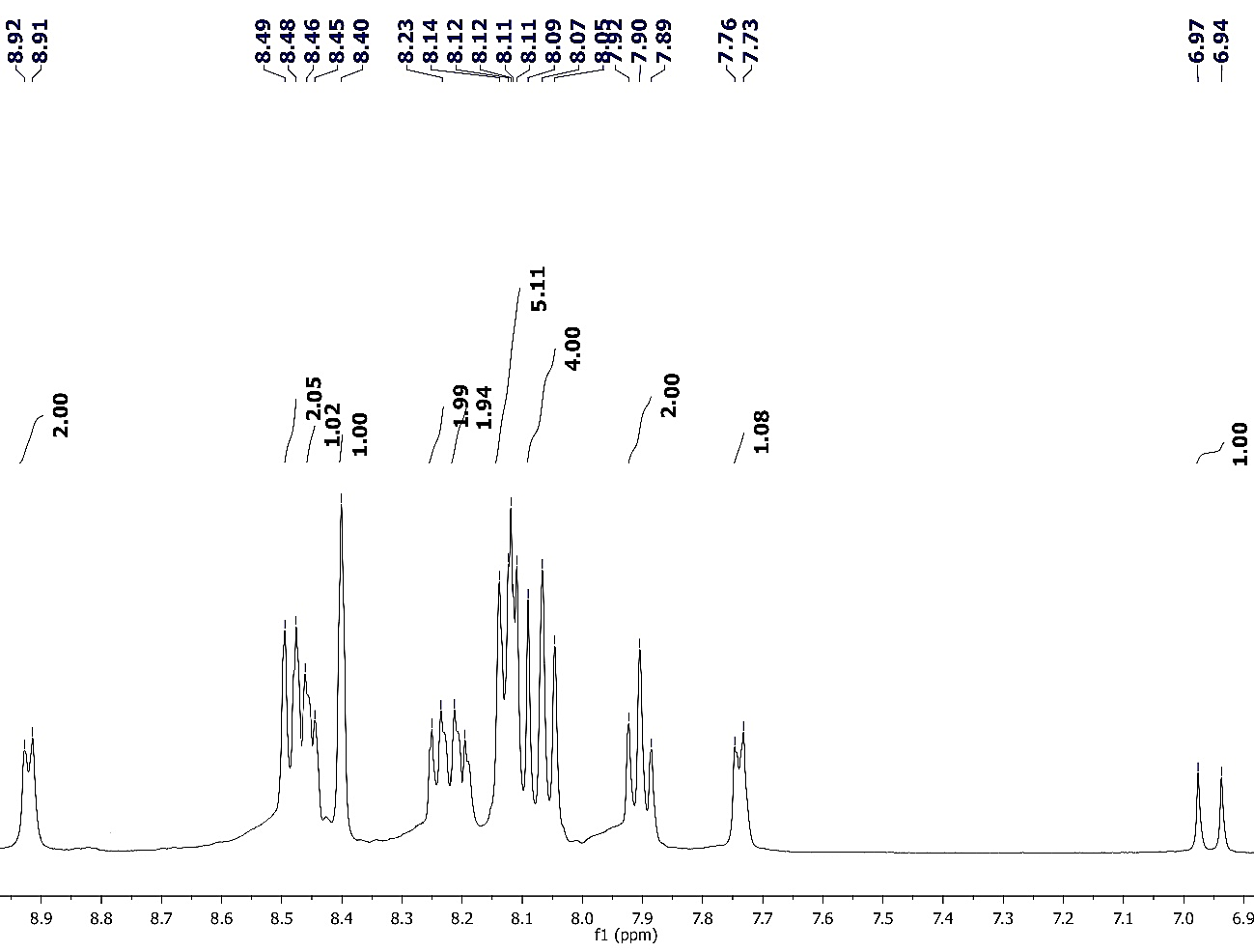

**Figure S12. ^1^H NMR spectrum of sensitizer SFA-8.**

**
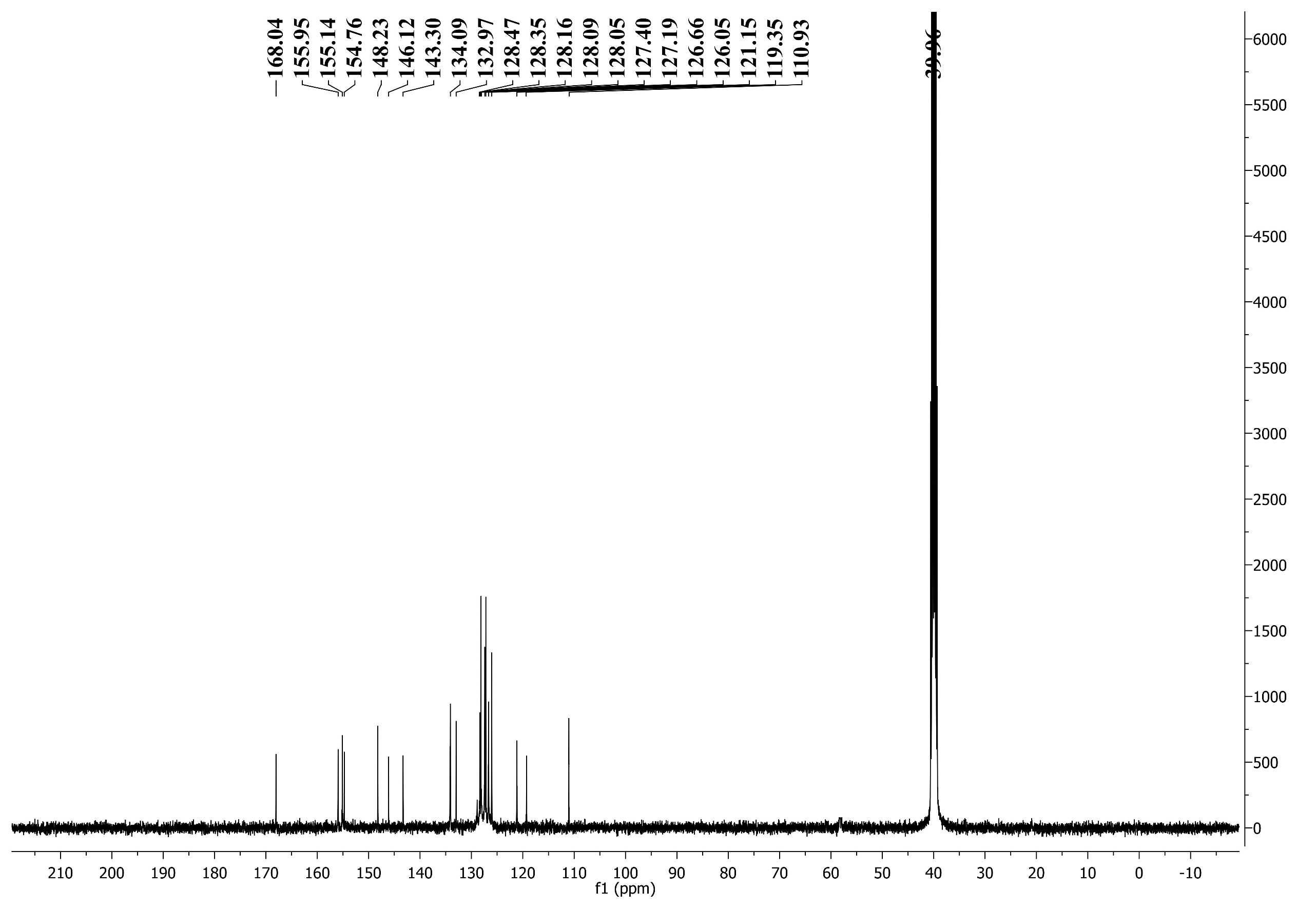
**

**Figure S13. ^13^C NMR spectrum of sensitizer SFA-8.**

**2. Cell preparations and photovoltaic characterizations**

2.1. TiO_2_ electrode preparation

A double-layer TiO_2_ photoelectrode (10 + 5) μm in thickness with a 10 μm thick nanoporous layer and a 5 μm thick scattering layer (area: 0.25 cm^2^) was prepared using a reported method. Fluorine-doped tin oxide (FTO) coated glasses (Nippon Sheet Glass Co., Japan) with a sheet resistance of 8-10 ohm-2 and an optical transmission of greater than 80% in the visible range were screen printed using anatase TiO_2_ colloids (particle size ∼ 13 nm) obtained from commercial sources (Ti-Nanoxide D/SP, Solaronix). Nanocrystalline TiO_2_ thin films were deposited onto the conducting glass by screen-printing, which was then sintered at 500 °C for 1 h. The film thickness was measured with a Surfcom 1400A surface profiler (Tokyo Seimitsu Co. Ltd.). The electrodes were impregnated with a 0.05 M titanium tetrachloride solution and sintered at 500 °C. The films were further treated with 0.1 M HCl(aq) before examination.

The dye solutions of the main sensitizer N719 (0.2 mM) were prepared in 1:1:1 mixture of acetonitrile, tert-butyl alcohol and dimethyl sulfoxide (DMSO). For co-sensitization, of S with **N719** (0.2 mM of each **SFA-5-8** + 0.2 mM **N719**) were dissolver in 1:1:1 mixtures of the same solvent used later. Deoxycholic acid was added to the dye solution as a coadsorbent at a concentration of 20 mM. The electrodes were immersed in the dye solutions and then kept at 25 ° C for 20 h to adsorb the dye onto the TiO_2_ surface.

For preparing the counter electrode, pre-cut TCO glasses were washed with water followed by 0.1M HCl in EtOH, and sonication in acetone bath for 10 min. These washed TCO were then dried at 400 °C for 15 min. Thin layer of Pt-paste (Solaronix, Platisol T/SP) on TCO was printed and the printed electrodes were then cured at 450 °C for 10 min.

2.2. Fabrication of dye-sensitized solar cell

Photovoltaic and incident photon-to-current efficiency (IPCE) measurements were made on sandwich cells, which were prepared using TiO_2_ coated working electrodes and platinum coated counter electrodes and were sealed using a 40 μm Syrlyn spacer through heating of the polymer frame. The redox electrolyte (Solaronix, Iodolyte HI-30) consisted of a solution of 0.6 M DMPII, 0.05 M I_2_, 0.1 M LiI and 0.5 M TBP in acetonitrile.

2.3. Photovoltaic measurements

Photovoltaic measurements of sealed cells were made by illuminating the cell through the conducting glass from the anode side with a solar simulator (WXS-155S-10) at AM 1.5 illuminations (light intensity: 100 mW cm^−2^).

2.4. Incident photon to current efﬁciency (IPCE) conversion

IPCE measurements were made on a CEP-2000 system (Bunkoh-Keiki Co. Ltd.). IPCE at each wavelength was calculated using Equation 1, where *I_SC_* is the short-circuit photocurrent density (mA. cm^−2^) under monochromatic irradiation, q is the elementary charge, λ is the wavelength of incident radiation in nm and P0 is the incident radiative flux in W/m^2^.

$$\mathrm{IPCE}\left( \lambda\right)=1240\left( \frac{I_{\mathrm{SC}}}{q\lambda P_{o}} \right) (\mathbf{1})$$

2.5. Electrochemical impedance spectroscopy (EIS)

The electrochemical impedance spectra were measured with an impedance analyzer potentiostat (Bio-Logic SP-150) under illumination using a solar simulator (SOL3A, Oriel) equipped with a 450 W xenon lamp (91160, Oriel). EIS spectra were recorded over a frequency range of 100 mHz to 200 kHz at room temperature. The applied bias voltage was set at the *V_OC_* of the DSSCs, with AC amplitude set at 10 mV. The electrical impedance spectra were fitted using Z-Fit software (Bio-Logic).

2.6. Cyclic voltammetry

Cyclic voltammetry (CV) was performed in DMF with the electrolyte 0.1 M [TBA][PF_6_] at a scan rate of 50 mV s^−1^. The working electrode used is the Glassy carbon, Pt wire represented the counter electrode and the reference electrode is Ag/Ag^+^ in ACN. Fc/Fc^+^ was introduced as internal reference.

**3. Molecular Modeling**

Equilibrium molecular geometries of **SFA-5-8** calculated using the Becke's three parameter hybrid functional, Lee–Yang–Parr's gradient corrected correlation functional (B3LYP) and (6-311g(d, p)) [1, 2, 3, 4]. The geometry optimization calculations were followed by energy calculations using time-dependent density functional theory (TD-DFT) utilizing the energy, functional B3lyp and the basis set 6-311g (d, p). The solvent (DMF) effect was accounted for by using the conductor-like polarizable continuum model (C-PCM), implemented in Gaussian 09.

**References**

[1] G. Melikian, F. Rouessac, C. Alexandre, Synth Commun. 1993, 23, 2631

[2] A. D. Becke, Phys. Rev. A **1988**, 38, 3098.

[3]C. T. Lee, W.T. Yang, R.G. Parr, Phys. Rev. B. **1988**, 37, 785

[4] N. Godbout, D.R. Salahub, J. Andzelm, E. Wimmer, Optimization of Gaussian-type basis-sets for local spin-density functional calculations .1. Boron through neon, optimization technique and validation. Can. J. Chem.-Rev. Can. Chim. **1992**, 70, 560-571.
